# China’s demographic dividend has moved from age-based labor supply to skill-based productivity

**DOI:** 10.1073/pnas.2532906123

**Published:** 2026-04-08

**Authors:** Hengyu Gu, Yingju Wu, Guillaume Marois, Wolfgang Lutz, Tianlong Niu

**Affiliations:** ^a^School of Geography and Ocean Science, Nanjing University, Nanjing 210023, China; ^b^Asian Demographic Research Institute, Shanghai University, Shanghai 200444, China; ^c^Wittgenstein Centre for Demography and Global Human Capital (University of Vienna, International Institute for Applied Systems Analysis, Vienna Institute of Demography/Austrian Academy of Sciences), International Institute for Applied Systems Analysis, Laxenburg 2361, Austria

**Keywords:** age support ratio, human capital, skill composition, economic growth

## Abstract

Accelerated global population aging challenges conventional economic growth paths. Yet, the mechanisms underlying the transition from the age-based demographic dividend, derived from a favorable age support ratio (ASR), to a skill-based dividend, driven by human capital accumulation, remain insufficiently understood. Using population census data for 336 Chinese cities from 2000 to 2020, we develop a task-based skill ratio (TSR) index to quantify the skill composition of local labor markets, capturing the relative intensity of high- and low-skill tasks within city-level employment structures. We identify a divergence trend where the ASR peaked around 2010 and has since declined, while the TSR has continued to rise and diffuse geographically. We further examine a synergistic effect between ASR and TSR on economic growth and project the compensatory TSR required under alternative demographic scenarios to 2100. It shows that both ASR and TSR positively affect the per capita GDP of a city, but the latter plays a dominant role. A higher ASR amplifies the economic returns to TSR, with the old-age support ratio (OSR) as the binding constraint. Projections indicate that delayed retirement can partly alleviate the effects of the ASR decline, but cannot reverse the long-term trend. Continued improvement in the TSR is therefore necessary to offset this structural demographic shift. Economic growth relies less on favorable age structures and increasingly depends on the skill composition of the workforce, making skill upgrading central to sustained prosperity.

## Significance

Global population aging is reducing the age-based demographic dividend that has long supported economic growth. This study identifies the skill-based dividend as the pivotal driver of future growth and highlights a mechanism where the economic returns to skills are amplified by a supportive age structure. This offers a critical insight for both developing nations approaching the end of their demographic window and developed nations already grappling with aging: While delayed retirement provides necessary mitigation, the fundamental path to sustaining economic prosperity lies in prioritizing skill enhancement.

The demographic transition, characterized by a decline in mortality followed by a reduction in fertility, creates a demographic dividend that has catalyzed economic growth miracles, particularly in East Asia (1, 2). This effect arises when the demographic structure shifts such that the productive population accounts for a larger share of the total (3–5). However, the subsequent “silver wave” of global aging is undermining the potential to achieve sustained prosperity (6–9). Demographic pressure is intensifying globally. For instance, between 2010 and 2023, 74 countries saw their age support ratios (ASRs), defined as the ratio of the working-age population (aged 15 to 64) to the non-working-age population, decrease by over 10% (10).

This demographic pressure, initially observed in developed nations, is now increasingly affecting emerging economies, particularly China (11, 12). While China successfully leveraged its historically favorable ASR to fuel decades of economic growth, this age-based advantage has been diminishing since 2010 amid accelerated aging and fertility decline (13, 14). Moreover, the ASR shows profound geographical differentiation among prefecture-level cities (encompassing both urban and rural areas), which has been significantly reshaped by internal migration (15). While megacities (e.g., Beijing and Shanghai) may sustain higher ASRs through the inflow of young workers, small and medium-sized cities face even more severe demographic challenges. In addition to the total population decrease of 3.39 million in 2025 (16), the deepening of an aging society has constrained the supply of young migrants (17), leading to the trend of demographic contraction. Thus, identifying pathways to transition from age-based labor supply to skill-based productivity is crucial to offset the waning age-based demographic dividend and secure the realization of Sustainable Development Goal (SDG) 8, namely promoting decent work and economic growth.

The prevailing narrative links the shifting demographic age structure to economic growth dynamics (18–20). Bloom and Williamson (1) emphasize that a higher ASR provides the foundation for economic growth. As regions transition into an aging society, the ensuing decline in ASR has been demonstrated to impede economic performance (13, 19, 21, 22). Rees et al. (17) utilize a multiregional demographic projection model to reveal the supply-side constraints that aging imposes on the quantity of the regional labor force. Lutz et al. (23) highlight the role of the education dividend, which shifts focus to the accumulation of human capital, aligning with the classical human capital theory (24). The underlying rationale is that a better-educated workforce possesses the skills required to create value in the knowledge economy (25, 26), thereby driving higher productivity. This implies that improving labor skills offers a quality-quantity substitution to offset the decline in workforce numbers (27, 28).

However, using educational attainment as a proxy for human capital may overlook the mismatch between skill supply and realized productivity (29, 30). Distinct from the input of formal schooling, human capital embodies the realized stock of skills required to drive economic growth (31). In China, the rapid expansion of higher education has precipitated significant credential inflation (32), meaning that years of schooling overestimate the actual productivity of the workforce. Besides, China’s unique household registration (*Hukou*) system acts as an institutional barrier that may impede high-skilled migrants from accessing job opportunities commensurate with their qualifications in megacities (33, 34). Thus, traditional metrics of educational attainment may fail to capture full productivity potential.

Labor skills constitute the foundation of human capital (35). The logic of labor market competition is shifting from educational thresholds to specific skills (36). Technological change has driven a pronounced skill polarization within the workforce, creating a distinction between high-skill and low-skill groups (37). Previous studies demonstrate that labor markets are increasingly shifting toward high skills, which enhances productivity (35, 38, 39). However, they are largely confined to the skill endowments of individual workers, and the aggregation of these microattributes into an indicator at the city level remains relatively underexplored. Driven by the industrial division of labor, the geographical differentiation in occupational compositions interacts with intercity functional linkages to shape different skill structures (40). We construct the task-based skill ratio (TSR) to quantify the skill composition of local workforces at the city-level (TSR, see *Data and Methods* for further details on the measurement of TSR).

This paper investigates how the age-based dividend is being transformed into a skill-based dividend in contemporary China. It addresses three questions. First, how can the skill composition of local labor markets be measured in the absence of occupational skills data in Chinese census sources? Second, how do changes in the ASR and TSR interact in shaping economic growth across cities? Third, to what extent can continued improvements in skill composition compensate for the long-term decline in the ASR under future demographic scenarios?

To answer these questions, we construct the TSR by adapting the framework of the U.S. Occupational Information Network (O*NET), which deconstructs occupations into measurable work activities (*SI Appendix*, Text 2). By assigning task-based skill scores to Chinese occupations and aggregating them to the city level (*SI Appendix*, Fig. S1, Step 1), we generate a consistent measure of local skill composition. Using this dataset, we find that while the national ASR reached a turning point around 2010 and subsequently declined, the TSR continued to rise and diffused beyond eastern megacities. We then estimate the marginal and interactive effects of ASR and TSR on per capita GDP (*SI Appendix*, Fig. S1, Steps 2 to 3) and project the TSR levels required to compensate for demographic aging from 2025 to 2100 (*SI Appendix*, Fig. S1, Step 4). This study clarifies the evolving demographic foundations of economic growth in aging societies by integrating a measurement strategy with a dynamic projection framework.

## Data and Methods

### Data Sources and Sample Construction.

This study employs a composite dataset that integrates urban macroeconomic indicators from the China City Statistical Yearbook and demographic data from China’s National Censuses (2000, 2010, 2020) and intercensal surveys (2005, 2015). Projected Data for the ASR from 2025 to 2100 are sourced from the United Nations World Population Prospects 2024 (10). Our initial investigation covers 336 Chinese cities from 2000 to 2020, accounting for over 98% of the national population (*SI Appendix*, Text 1). For empirical estimation, the sample is restricted to a balanced panel of 289 cities to exclude missing control variables, a refinement that maintains coverage of over 90% of the national population.

### Measurement of the ASR.

The ASR can serve as a proxy for the demographic window of opportunity. Consistent with existing research and the statistical standards of the National Bureau of Statistics of China (23, 41–43), we construct this indicator by classifying the resident population into three age groups: children ($Pop_{0-14}$), the working-age population ($Pop_{15-64}$), and older adults ($Pop_{65+}$). Based on this classification, the ASR for city c in year t is defined as

[1] $$ASR_{\mathrm{ct}}=\frac{Pop_{15-64,ct}}{Pop_{0-14,ct}+Pop_{65+,ct}},$$

where $Pop_{age,ct}$ represents the population of the respective age groups in city c at time t.

### Decomposition of the ASR.

To distinguish the generational sources of demographic support potential, we decompose the ASR into two subindicators, the youth support ratio (YSR) and the old-age support ratio (OSR). The children represent the foundational source of future labor skills and the plasticity of human capital accumulation, making the YSR a proxy for the demographic support base available to the children. The older adults have largely exited the labor market with fixed skill stocks (17), making the OSR a measure of the demographic support strength for the older adults (44). The indicators are defined as follows:

[2] $$YSR_{\mathrm{ct}}=\frac{Pop_{15-64,ct}}{Pop_{0-14,ct}},$$

[3] $$OSR_{\mathrm{ct}}=\frac{Pop_{15-64,ct}}{Pop_{65+,ct}}.$$

### Occupational Cross-National Matching and TSR Quantification.

Given the absence of granular occupational data in China, we design a cross-national occupational matching framework for TSR quantification (detailed in *SI Appendix*, Text 2). Premised on the cross-national consistency of technical divisions of labor in modern industrial systems, this framework adapts the O*NET “Work Activities” data to quantify occupational skill profiles in China (37). Task attributes are categorized into high-skill (e.g., creative thinking), low-skill (e.g., machine control), and neutral components (*SI Appendix*, Table S6), using a “Level” to measure the skill intensities. To ensure the robustness of the matching process, we implement semantic matching powered by large language models (LLMs) to align 347 Chinese National Occupational Classification codes with O*NET versions 5.0 through 27.2, thereby capturing dynamic, time-specific skill evolutions over the study period.

To align with the granularity of census data and mitigate potential biases from cross-national classification mismatches, we aggregate 3-digit occupational skill scores to the 2-digit level. Measured on a 7-point scale, the high-skill intensity scores ($h_{j}$) across the 2-digit occupational groups range from 1.74 to 5.38 (SD = 0.89), while low-skill intensity scores ($l_{j}$) range from 1.74 to 4.11 (SD = 0.65), reflecting heterogeneity in task composition (*SI Appendix*, Fig. S7). The city-level TSR is defined as the ratio of high-skill task content within the local workforce compared to low-skill task content. Specifically, it is operationalized as the ratio of aggregate high-skill to low-skill intensities, both weighted by employment. The TSR can be written as

[4] $$TSR_{\mathrm{ct}}=\frac{\sum_{j=1}^{J}\;(E_{\mathrm{jct}}\times h_{j})}{\sum_{j=1}^{J}\;(E_{\mathrm{jct}}\times l_{j})},$$

where $E_{\mathrm{jct}}$ represents the employment share of occupation j in city c at time t.

### Two-Way Fixed Effects Model (TWFEM).

We construct a TWFEM that controls for city and temporal effects to identify the relationship between economic level, age-based dividend, and skill-based dividend. Chinese cities exhibit significant geographical differences and temporal trends. This specification is essential to absorb unobserved time-invariant characteristics that would otherwise bias our estimates. Eq. **5** is the baseline specification to estimate the independent effects of ASR and TSR. Building on this benchmark, Eq. **6** introduces the interaction term to further examine the synergistic effects of age-based and skill-based dividends on economic outcomes. The specific model is as follows:

[5] $$\begin{array}{ll} \ln(\text{GDP}\;\text{per}\;{\text{captia}}_{ct}) & =\beta_{0}+\beta_{1}{\text{ASR}}_{ct}+\beta_{2}{\text{TSR}}_{ct}\\ & +\gamma X_{ct}+\mu_{c}+\lambda_{t}+\varepsilon_{ct}, \end{array}$$

[6] $$\begin{array}{ll} \ln(\text{GDP}\;\text{per}\;{\text{captia}}_{ct}) & =\beta_{0}+\beta_{1}{\text{ASR}}_{ct}+\beta_{2}{\text{TSR}}_{ct}\\ & +\beta_{3}({\text{ASR}}_{ct}\times {\text{TSR}}_{ct})\\ & +\gamma X_{ct}+\mu_{c}+\lambda_{t}+\varepsilon_{ct}, \end{array}$$

where $\ln({\text{GDP per capita}}_{\mathrm{ct}})$ denotes the natural logarithm of GDP per capita of city c in year t; ${\text{ASR}}_{\mathrm{ct}}$ and ${\text{TSR}}_{\mathrm{ct}}$ denote the ASR and TSR, respectively; $X_{\mathrm{ct}}$ is a vector of control variables; $\mu_{c}$ and $\lambda_{t}$ capture city- and year-fixed effects; and $\varepsilon_{\mathrm{ct}}$ is the error term. We select a vector of control variables $X_{\mathrm{ct}}$, to satisfy the conditional independence assumption (45). This specification aims to simultaneously eliminate confounding bias arising from regional heterogeneity and absorb residual variance to enhance estimation precision (detailed in *SI Appendix*, Table S5). Furthermore, we substitute the aggregate ASR with the YSR and OSR in subsequent decomposition analyses.

### Dynamic Projection of Scenarios.

To quantify the TSR enhancement required to counteract demographic pressure, we construct a dynamic projection based on the United Nations’ Low Fertility Variant (2025–2100). We opt for the low variant because recent fertility trends in China are well below the medium-level projections, making the low variant a more realistic reflection of China’s population dynamics. To project three retirement policy scenarios under this baseline, we define the working-age population age ranges as 15 to 64, 15 to 69, and 15 to 74, respectively (17, 43). All ASR indicators are standardized to the 2000–2020 baseline for consistency (detailed in the *SI Appendix*, Text 3). Based on the principle of maintaining the net demographic contribution C (defined in *SI Appendix*, Eq. **S4**), the compensatory $TSR_{t,comp}^{*}$ is derived as

[7] $${\mathrm{TSR}}_{t,comp}^{*}=\frac{C-\beta_{1,t}ASR_{t}^{*}}{\beta_{2,t}+\beta_{3}ASR_{t}^{*}},$$

where $ASR_{t}^{*}$ denotes the projected ASR under the specific retirement scenario at year t; ${\mathrm{TSR}}_{t,comp}^{*}$ represents the requisite TSR level to maintain the baseline contribution by counterbalancing the scenario-specific projection of $ASR_{t}^{*}$ in a given future year t; $\beta_{3}$ is the interaction term, and $\beta_{1,t}$ and $\beta_{2,t}$ denote the coefficients for ASR and TSR, respectively. These parameters originate from the empirical estimates of Eq. **6** but follow dynamic paths (±1% annual change) to project evolution. We enforce an “effectiveness floor” (ϵ=0.01) on the marginal return of TSR to preclude mathematical singularity in extreme contexts (detailed in *SI Appendix*, Text 3.4).

## Results

### The Decline and Geographical Differentiation of the ASR.

**Fig. 1.**
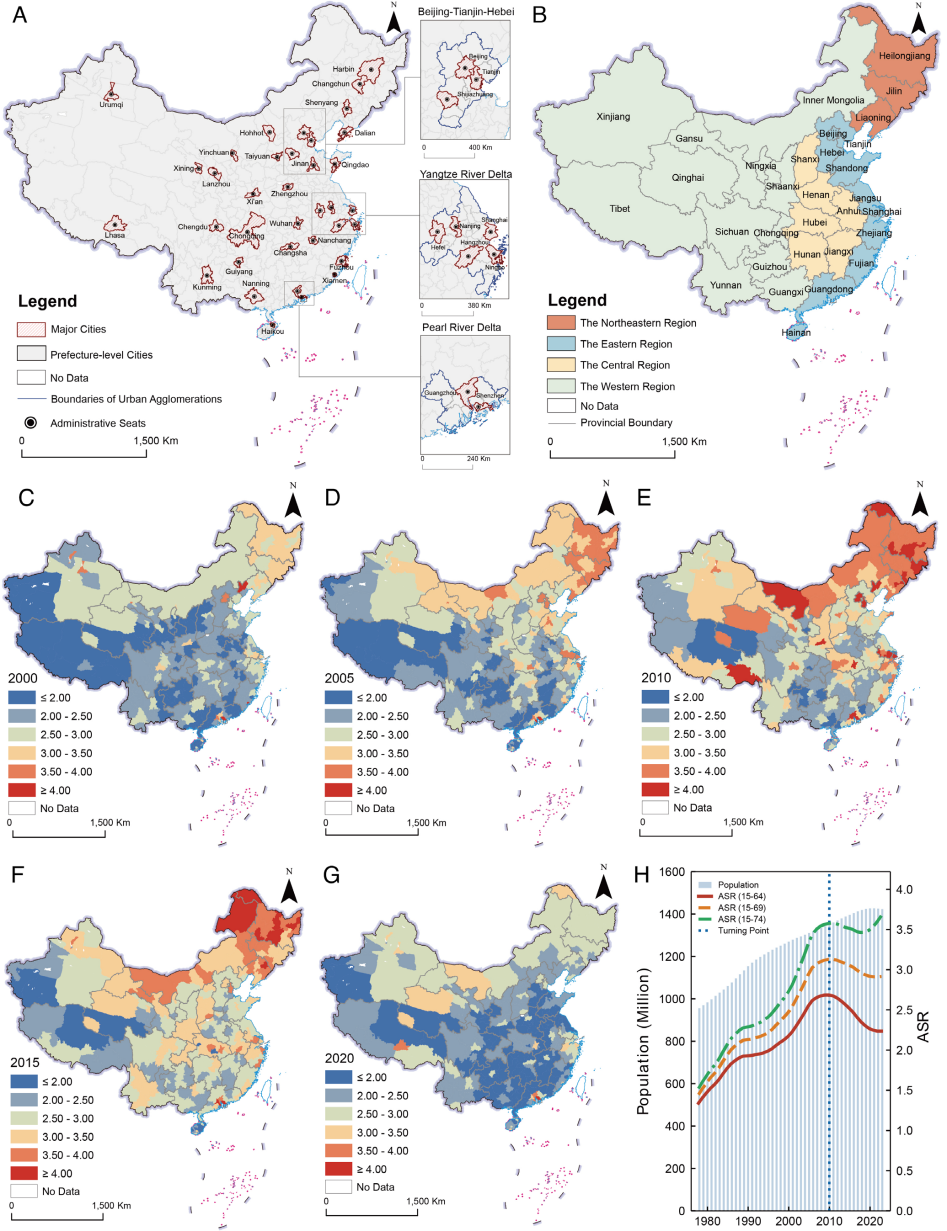
Geographical pattern of ASR. (*A*) shows the geographical distribution of major cities and urban agglomerations in China. (*B*) illustrates the classification of the four major economic regions used for comparative analysis. (*C*–*G*) display the city-level ASR distribution from 2000 to 2020. (*H*) presents the national trends in total population and ASRs calculated for working-age populations defined as age groups 15 to 64, 15 to 69, and 15 to 74 from 1978 to 2023.

National trends reveal a turning point in China’s ASR around 2010. From 1978 to 2010, the national ASR climbed steadily from 1.34 to a peak of 2.69 (Fig. 1*H*), marking the onset of a demographic window (46). However, since 2010, the ASR has entered a sustained decline at an average annual rate of 1.4%. This trend marks the erosion of the age-based labor supply advantage. The decline intensified after 2015, which indicates that the age-based dividend is diminishing due to the deepening challenges of population aging. Notably, when the working-age population is redefined as the 15 to 74 age group, the ASR exhibits a resurgence around 2020. This upward trend suggests that extending the retirement age to 75 may serve as an effective strategy to alleviate the pressure on the standard labor supply (43).

We analyze demographic patterns across 336 cities using a multiscalar framework spanning four major economic regions, major urban agglomerations, and major cities (Fig. 1 *A*–*B*). The visualization illustrates a significant geographical differentiation in age support potential (Fig. 1 *C*–*G*). During the period from 2005 to 2015, the Northeastern region maintained higher ASR clusters. This pattern stemmed from the region’s earlier demographic transition, which generated a large working-age population (46). After 2015, a pervasive decline in ASR occurred across all regions. This nationwide downward shift is further highlighted in population cartograms (*SI Appendix*, Fig. S2). Despite this decline, a few cities within major urban agglomerations, particularly the Pearl River Delta (PRD) and Yangtze River Delta (YRD), have maintained robust ASR levels by absorbing migrants (47).

### The Rise and Spatial Diffusion of the TSR.

**Fig. 2.**
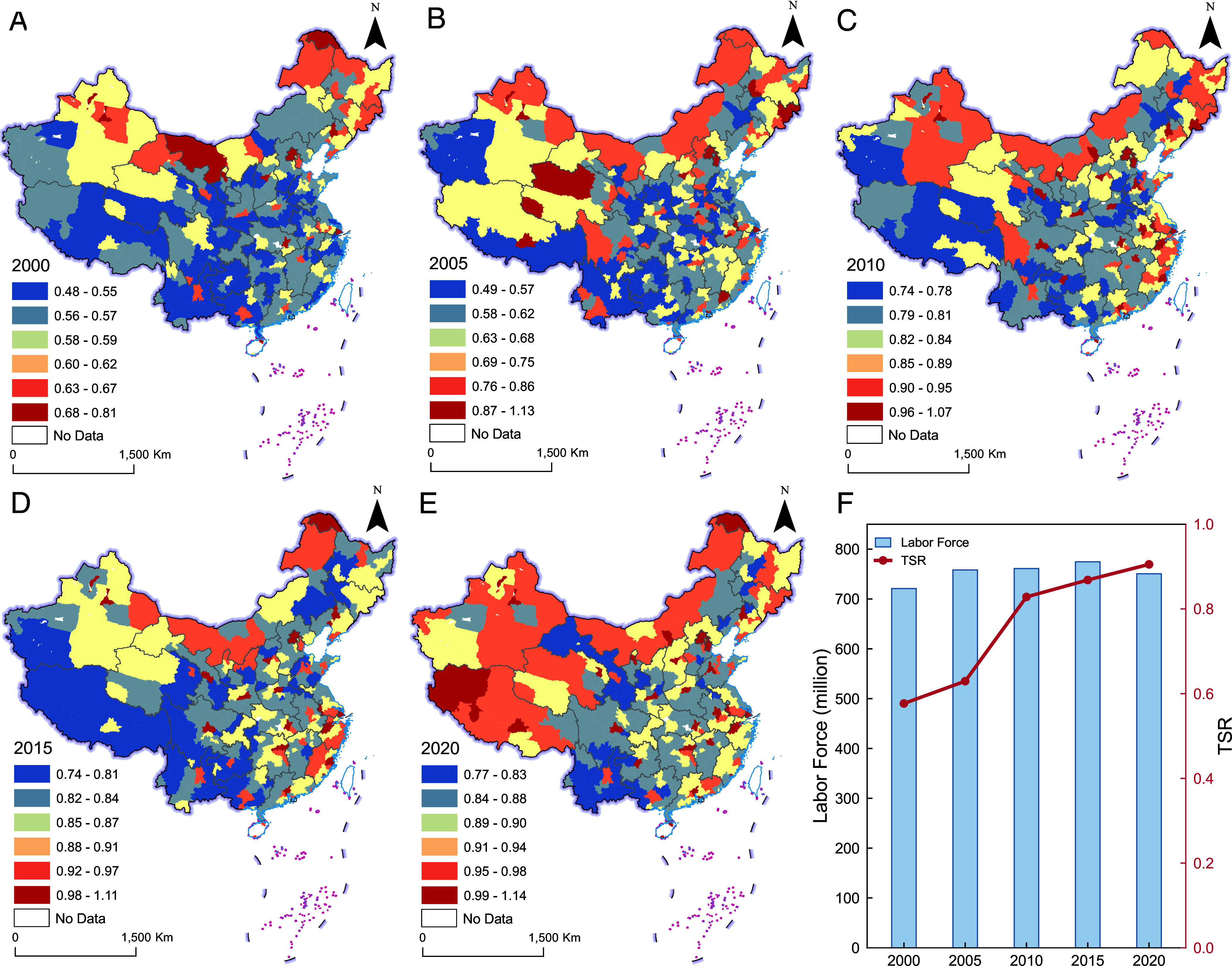
Geographical pattern of TSR. (*A*–*E*) depict the city-level distribution of the TSR across Chinese cities from 2000 to 2020. (*F*) presents the overall trend of the skilled labor force at the national level. Due to data limitations, the skilled labor force data are available for only five periods.

In contrast to the turning point of the ASR, the national average TSR maintained steady growth at an average annual rate of 2.26% from 2000 to 2020 (Fig. 2*F*). This trend indicates that despite the decline of the working-age population, the skill composition of the workforce is shifting toward high-skill tasks.

The distribution of TSR evolved from a concentration in eastern megacities to a diffusion into inland major cities. From 2000 to 2010, high TSR values were primarily clustered in eastern megacities (Fig. 2 *A*–*C*). Despite the pressure of declining ASR, the TSR began diffusing to inland major cities after 2010. The rapid rise of regional centers (e.g., Chengdu and Wuhan) fostered a more multipolar geographical pattern (Fig. 2 *D* and *E*). This transition toward a more balanced skill distribution is validated by population cartograms (*SI Appendix*, Fig. S3). However, this diffusion has not diminished the dominance of top-tier cities: Megacities like Beijing continued to maintain exceptional skill intensity (*SI Appendix*, Fig. S4).

### Economic Impacts of ASR and TSR.

The divergence between declining age support and rising skill intensity raises a question: How do these structural shifts translate into economic performance? To estimate the marginal effects of each, we utilize a TWFEM linking ASR and TSR to per capita GDP. Column 1 of Table 1 confirms an asymmetry in the effects of ASR and TSR on economic development: The standardized regression coefficient for TSR is 0.088 (P<0.01), an effect size approximately 2.4 times that of ASR ($\beta_{1}=0.036,\;P<0.01$). This finding remains robust after addressing potential endogeneity using an instrumental variable approach (*SI Appendix*, Table S1). We further validate the influence of ASR and TSR using AI classifications, conventional indicators, and fixed O*NET base years, confirming that the result holds (*SI Appendix*, Table S8).

**Table 1.** Regression estimates for ASR and TSR effects on per capita GDP

	Age support ratio		Youth support ratio		Old-age support ratio	
	(1)	(2)	(3)	(4)	(5)	(6)
ASR	0.036^***^ (0.009)	0.039^***^ (0.009)				
TSR	0.088^***^ (0.020)	0.090^***^ (0.020)	0.090^***^ (0.020)	0.091^***^ (0.020)	0.087^***^ (0.019)	0.089^***^ (0.019)
ASR×TSR		0.017^***^ (0.006)				
YSR			0.014 (0.011)	0.008 (0.012)		
YSR×TSR				0.006 (0.006)		
OSR					0.030^*^ (0.018)	0.040^**^ (0.017)
OSR×TSR						0.022^**^ (0.009)
Ln Pcinvest	0.054^***^ (0.015)	0.062^***^ (0.015)	0.054^***^ (0.015)	0.057^***^ (0.015)	0.055^***^ (0.015)	0.060^***^ (0.015)
Ln Patent	0.020^***^ (0.006)	0.019^***^ (0.006)	0.022^***^ (0.006)	0.021^***^ (0.006)	0.018^***^ (0.006)	0.019^***^ (0.006)
Ln Population	−0.834^***^ (0.047)	−0.856^***^ (0.046)	−0.820^***^ (0.048)	−0.827^***^ (0.048)	−0.841^***^ (0.047)	−0.863^***^ (0.048)
Ln Road	−0.007 (0.019)	−0.002 (0.019)	−0.009 (0.019)	−0.008 (0.019)	−0.009 (0.019)	−0.003 (0.019)
Ln Newfirms	0.024^**^ (0.009)	0.024^**^ (0.009)	0.026^***^ (0.009)	0.027^***^ (0.010)	0.027^***^ (0.009)	0.022^**^ (0.010)
Ln Fdi	0.015^***^ (0.004)	0.016^***^ (0.004)	0.015^***^ (0.004)	0.016^***^ (0.004)	0.015^***^ (0.004)	0.016^***^ (0.004)
Tertiaryshare	−0.003^***^ (0.001)	−0.003^***^ (0.001)	−0.004^***^ (0.001)	−0.004^***^ (0.001)	−0.004^***^ (0.001)	−0.004^***^ (0.001)
Constant	12.969^***^ (0.388)	12.951^***^ (0.378)	12.889^***^ (0.403)	12.872^***^ (0.399)	12.963^***^ (0.392)	13.045^***^ (0.384)
City and Year FE	Yes	Yes	Yes	Yes	Yes	Yes
Observations	1,445	1,445	1,445	1,445	1,445	1,445
R^2^	0.981	0.981	0.980	0.980	0.980	0.981

*Note:* SE are clustered at the city level and reported in parentheses. *, **, and *** denote statistical significance at the 10%, 5%, and 1% levels, respectively. To facilitate the comparison of regression coefficients, all key independent variables, including ASR, YSR, OSR, and TSR, have been standardized using the equation $Z_{\mathrm{it}}=\left(X_{\mathrm{it}}-\mu \right)/\sigma$, where μ and σ denote the global mean and SD of the corresponding variable, respectively. FE refers to fixed effects, with both city and year fixed effects included in all models to control for unobserved heterogeneity.

**Fig. 3.**
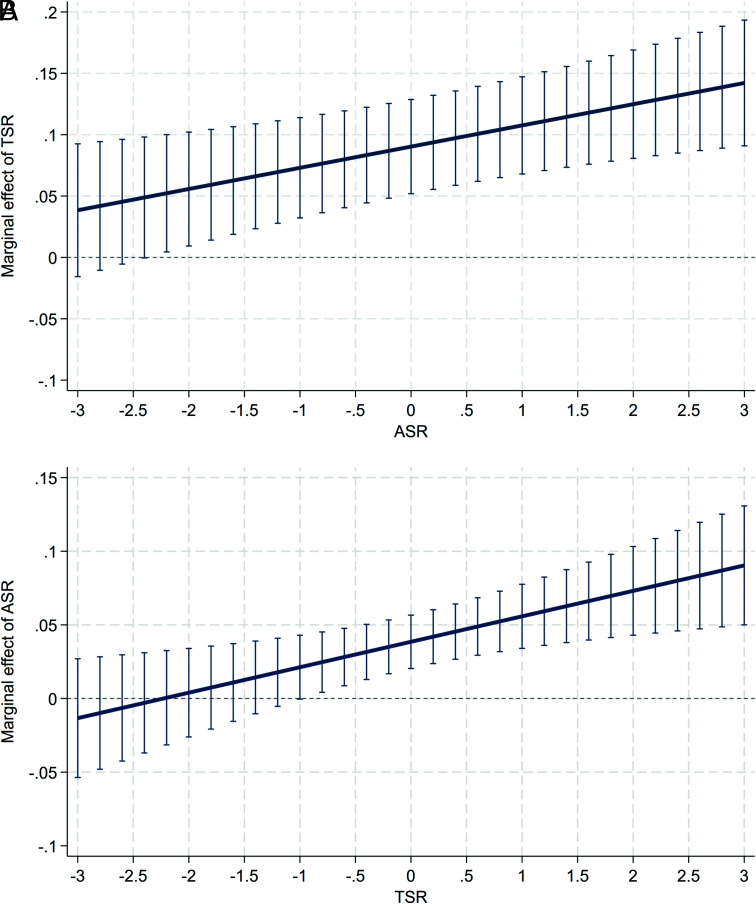
Synergistic effects of ASR and TSR on economic development. (*A*) displays the marginal effect of TSR as a function of ASR, defined by the partial derivative $\beta_{2}+\beta_{3}\times {\text{ASR}}_{\mathrm{ct}}$ based on Eq. **6**. (*B*) displays the marginal effect of ASR as a function of TSR, defined as $\beta_{1}+\beta_{3}\times {\text{TSR}}_{\mathrm{ct}}$. Vertical lines denote 95% CI based on SE clustered at the city level. ASR and TSR are standardized to facilitate comparison.

The interaction analysis further reveals that a synergistic effect, a mutually reinforcing relationship where a higher level of one factor amplifies the economic returns to the other, governs the interplay between ASR and TSR. The significantly positive coefficient of the interaction term in Table 1 (Column 2, $\beta_{3}=0.017,P<0.01$) confirms this complementarity: a higher ASR amplifies the economic returns to TSR, and vice versa. While TSR drives growth even at relatively low ASR levels (Fig. 3*A*), ASR’s economic effect is conditional, becoming significant if TSR surpasses a critical threshold (standardized≈-1; raw ratio≈0.62) (Fig. 3*B*). It implies that a sufficient TSR is a prerequisite for unlocking the age-based dividend. In cities with a low-end TSR, expanding the size of the working age population does not translate into economic growth.

Columns 3 to 6 of Table 1, which decompose the ASR into YSR and OSR, reveal a divergence in how these two components interact with the TSR. The interaction between YSR and TSR is statistically insignificant, suggesting that the YSR has not constituted a binding constraint on the skill-based dividend, given that the youth population represents the foundation for future human capital accumulation (23, 48). The interaction between OSR and TSR is significantly positive ($\beta_{3}=0.022,\;P<0.05$), with a coefficient magnitude exceeding even that of the ASR. This constraint of OSR is driven by the withdrawal of older adults from the productive sector, a process inherently linked to skill obsolescence (49). Unlike the younger generation, accumulating human capital, older adults face a depreciation in adaptability to evolving task requirements (13). The decline in the OSR signifies not merely a reduction in labor supply but a loss of experienced human capital that lacks the flexibility to align with high-skill sectors, thereby impeding the full utilization of the growth potential offered by the TSR.

We further examine the associations of ASR and TSR with economic development across different city types. In small and medium-sized cities, we observe a positive interaction between ASR and TSR. Large cities, often the beneficiaries of preferential resource allocation, exhibit a nonsignificant interaction term but a substantial direct coefficient for TSR (*SI Appendix*, Table S2). This pattern is characterized by a strong dependence on high-skill intensity, reflecting the concentration of talent in metropolises (33, 47). Furthermore, stratifying cities by net migration volumes reveals the role of the *Hukou* system as an institutional screening barrier. We find that high net inflow cities display a significant synergistic association ($\beta_{3}=0.016,\;P<0.05$) (*SI Appendix*, Table S3). This suggests that *Hukou*-induced barriers tend to select for a younger and more mobile workforce, thereby clustering the demographic attributes necessary to activate the synergy between ASR and TSR. Conversely, low net inflow cities do not exhibit this statistical relationship. The above findings imply a spatially embedded transition: While high net inflow cities leverage selective migration to maximize synergistic returns, low net inflow cities rely more on the foundational support of their local demographic structure to realize skill-related economic potential.

### Three Phases of the Demographic-Skill Transition.

**Fig. 4.**
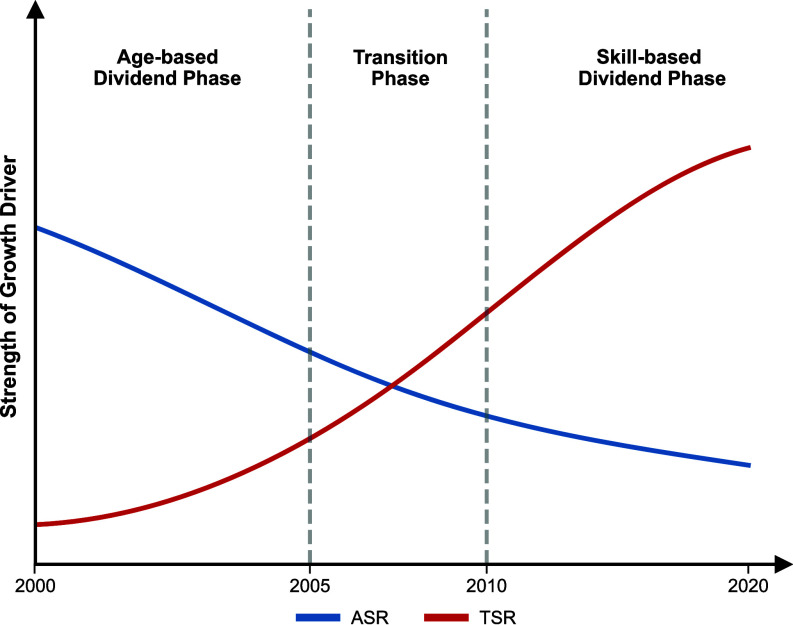
Three-Phase transition of China’s demographic dividend. This figure illustrates the structural transition of China’s demographic dividend over time. The Age-based Dividend Phase reflects economic growth primarily driven by the ASR. The Transition Phase marks a critical period of factor substitution, where the contribution of ASR diminishes while the impact of TSR accelerates, leading to a structural crossover. The Skill-based Dividend Phase is characterized by TSR driven growth, representing a regime where economic performance is sustained by skill accumulation. Solid curves illustrate the changing strength of each growth driver, while vertical dashed lines indicate the temporal boundaries between phases.

Our analysis identifies a transformation in the demographic foundations of China’s economic growth (Fig. 4). This periodization is supported by two independent, cross-validating lines of evidence: First, the time-varying marginal effects, which capture the change of ASR and TSR as growth drivers (*SI Appendix*, Fig. S5); second, the Gini coefficients, which quantify the trend of the spatial disparities around these two factors (*SI Appendix*, Fig. S6).

The Age-based Dividend Phase (c. 2000 to 2005). During this period, economic growth was primarily driven by the age-based dividend. Restrictive fertility policies accelerated the rise of the ASR, creating the window for the demographic dividend (46). China’s market-oriented reforms and the relaxation of *Hukou* constraints weakened administrative barriers to migration, catalyzing the massive transfer of low-cost labor from agriculture to industry.

The Transition Phase (c. 2005–2010). The marginal contribution of ASR experienced a substantial decline, converging with the rising influence of TSR (*SI Appendix*, Fig. S5). This convergence indicates that the growth pattern relying on labor quantity was reaching its limits. It is driven by the law of diminishing marginal returns: As labor-intensive industries became saturated, the economy’s capacity to absorb the demographic surplus derived from a high ASR diminished. Traditional labor-intensive industries became difficult to sustain high-speed development demands, resulting in a period of friction where the effects of ASR and TSR intersected and oscillated. This fundamental shift in growth logic is simultaneously evidenced by the trajectories of the Gini coefficient (*SI Appendix*, Fig. S6*K*).

The Skill-based Dividend Phase (post-2010). The long-run consequences of the restrictive fertility policies precipitated a decline in labor supply and diminished the marginal effect of ASR. Concurrently, the widespread industrial upgrading and the imperative for technological sophistication have propelled the TSR to become the primary driver of economic growth. This shift in dynamics is corroborated in the geographical pattern: The TSR-Gini remained high during this stage while the ASR-Gini fell. This demonstrates that a city’s economic standing has become more correlated with its TSR than with its ASR (*SI Appendix*, Fig. S6 *D−E*, *I*, and *J*).

Our above findings point to a conclusion: China’s growth dividend has not completely disappeared, but its source and realization mechanism have undergone an evolution. The demographic dividend founded on a higher ASR is indeed receding. However, a TSR defined by optimized skills is forming, which effectively counteracts the negative impacts of population aging by enhancing productivity. This endogenous transformation of growth drivers, from an extensive expansion reliant on labor-intensive production to an intensive development based on a “talent strategy”, also provides a powerful explanation for how China’s economy has maintained strong resilience amid rapid demographic aging. Therefore, this study offers a key insight for other countries facing demographic transitions: The sustainability of a growth dividend stems not from population size itself, but from the ability to effectively align improvements in human capital quality with the dynamic advantages of the ASR.

### Projection of Compensatory TSR Requirements.

Under the Low Fertility Variant baseline (defined as working-age 15 to 64 with static coefficients), the projection reveals that the pressure for TSR compensation shifts from a phase of mild fluctuation to one of accelerated accumulation. During the period of relative demographic stability (2025–2035), the required TSR exhibits slight fluctuations around the 2020 baseline. After 2050, as the economic consequences of population aging become more pronounced, the trajectory shifts into a steep upward trend. The TSR value climbs to 1.056 by 2050 and further accelerates to 1.48 by 2080. This projected 63% increase relative to the 2020 baseline implies that only substantial improvements in the TSR can preserve long-term economic momentum.

Extending the definition of the working-age population (to the 15 to 69 and 15 to 74 age groups) provides a mitigation that alters the required TSR rising gradient. When the upper boundary of the working age is extended to 69 and 74, the expansion of labor supply through delayed retirement dilutes the impact of ASR decline (9), causing a downward shift in the TSR compensation curve. In the 15 to 74 scenario, the required TSR in 2035 drops to 0.682, approximately 24.8% lower than the 2020 baseline, which opens a substantial window for adjustment in the medium term. Yet, while this expansion lowers the absolute level of demand, it does not reverse the long-term upward trend; by 2080, even under the 15 to 74 scenario, the TSR requirement rebounds to 1.023. This rebound indicates that delayed retirement alone can postpone but not eliminate the long-term pressure.

Beyond demographic adjustments, our analysis highlights that the evolution of economic parameters (β) exerts a decisive influence, driving a divergence in future outcomes that significantly outweighs the effects of delayed retirement. We consider scenarios where the marginal economic sensitivity to ASR intensifies by 1% annually, capturing the rising costs of labor scarcity. Under the 15 to 64 definition, the required TSR skyrockets to 1.844 by 2080, representing a 103.2% increase over the 2020 baseline. When this scarcity effect is compounded by diminishing skill returns, the required TSR surges to 2.761 by 2080, marking a 204% deviation from the baseline (*SI Appendix*, Fig. S10). Even with the most aggressive retirement extension (15 to 74), if the marginal effect of ASR continues to increase, the requirement rises by 23.7% over the baseline by 2080. This indicates that in a future characterized by increasing factor scarcity, expanding labor supply is insufficient to offset the economic pressures.

**Fig. 5.**
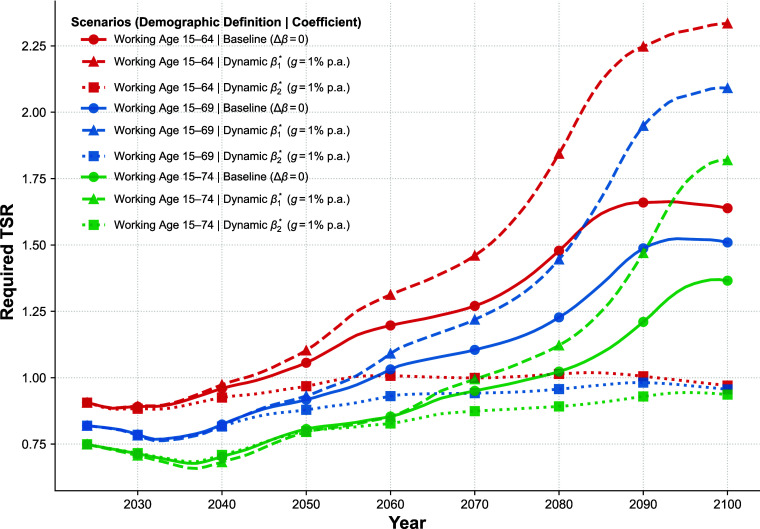
Projections of the Required TSR under alternative demographic and parameter scenarios. The projections simulate the TSR trajectories (2025–2100) by interacting three alternative definitions of the working-age population (setting the upper age limit at 65, 70, and 75 y) with three coefficient evolution settings: the static Baseline (Δβ=0), the Age-Dynamic scenario (annual 1% growth in the ASR coefficient $\beta_{1}$), and the Skill-Dynamic scenario (annual 1% growth in the TSR coefficient $\beta_{2}$).

In scenarios where the marginal return to TSR improves by 1% annually, the compensation curve is effectively flattened. The projection indicates that with sustained growth in skill returns, the required TSR for the 15 to 64 group in 2080 is contained at 1.01, stabilizing the long-term skill demand. Most notably, when the quantitative expansion of the “15 to 74” scenario is combined with the synergistic effect of the “Skill-Dynamic” scenario, the required TSR in 2080 falls to 0.892, which is 1.7% lower than the 2020 baseline. This result suggests that combining the enhancement of TSR with a moderate extension of working life may offer a viable path to navigate the challenge of deep aging (Fig. 5).

## Discussion

While the age-based advantage has contracted nationwide, persisting only in major urban agglomerations, the skill-based advantage has diffused hierarchically from coastal megacities to inland regional centers. Conventional wisdom holds that as the demographic window closes, the positive effects of ASR gradually fade, and it will cause a deceleration that exposes the Chinese economy to the risk of long-term stagnation (2, 48). Challenging this narrative of decline, our results demonstrate that the driver of China’s economic growth is shifting from a reliance on labor quantity to a development path driven by skill quality. This process is governed by a synergistic mechanism rather than a simple linear substitution. As a conditional amplifier, the ASR interacts with the TSR and magnifies the economic returns of skill accumulation, while the upgrading of the TSR serves as an activator that revitalizes the economic growth derived from the ASR.

The OSR, rather than the YSR, is the binding constraint on the skill-based dividend. This indicates that the synergistic mechanism is primarily driven by the retention of the productive-age population relative to older adults, reflecting the challenge of skill obsolescence associated with aging. This synergy is not uniform but is institutionally embedded. Megacities have effectively decoupled from age structure constraints, with their growth driven primarily by high-skill intensity independent of ASR. In contrast, small and medium-sized cities and high-net-inflow cities remain dependent on the synergistic interaction between ASR and TSR. For high-net-inflow cities, institutional screening (e.g., via the *Hukou* system) can reinforce rather than disrupt this synergy by selectively clustering a younger workforce. This process preserves the demographic foundation necessary for maximizing skill returns.

The synergistic effect not only explains China’s economic resilience but also offers a generalizable policy blueprint for nations across the demographic transition spectrum. For developing nations that are still benefiting from a demographic window, our findings offer a prescient guide. While a favorable ASR provides a transient advantage, the onset of population aging is an eventual certainty. This paper thus elucidates the imperative to proactively invest in the national TSR prior to this window’s closure, thereby transforming a period of demographic adjustment into a strategic opportunity for a leap in human capital.

For economies already contending with advanced aging, our research recasts the policy discourse. Conventional approaches often prioritize restoring demographic numbers through pronatalist incentives (8). Yet, escaping the low-fertility trap has proven difficult (50), and even successful interventions face a multidecade lag before newborns transform into productive labor. Similarly, while international migration has often been considered a demographic remedy, replacement migration on the scale required to stabilize age structures in large populations remains practically infeasible and socially unsustainable (17). Delayed retirement can partially mitigate the economic consequences of a declining ASR. However, our projections indicate that such adjustments to labor supply, while providing a medium-term buffer, cannot reverse the long-term drag of demographic pressures. This necessitates a dual shift: restructuring occupational composition toward high-value-added sectors to create a greater supply of high-skilled jobs and enhancing skill intensity within existing roles to enhance per-capita productivity.

The urgency of such a shift is already evident in current labor market dynamics. The rapid expansion of higher education has recently encountered challenges (32), evidenced by rising graduate unemployment and a subsequent drive toward vocational training. This phenomenon underscores that simply expanding educational attainment without matching task-based skill demands is insufficient; the focus must shift to realized productivity as captured by the TSR. Furthermore, the spatial diffusion of the skill-based dividend predicted by our framework is already manifesting. Recent trends show graduates migrating to inland major cities (34), a reallocation that not only alleviates the congestion costs in megacities but also injects vital productivity into regional hubs.

It is also important to acknowledge the limitations in our projection methodology. Although the marginal effects derived from our regressions are statistically significant and theoretically sound, their use in long-term forecasting may be constrained by the applicability of current estimates to future extrapolations. Nevertheless, the projection results grounded in the United Nations’ Low Fertility Variant have amply demonstrated the critical role of the TSR in addressing future economic growth pressures. Whether under the rigorous constraints of the standard retirement baseline (15 to 64) or the partially mitigated conditions of delayed retirement scenarios (15 to 69 and 15 to 74), the results consistently confirm a significant compensatory effect: Increasing the TSR is essential to offset the disadvantages of the age structure. The insights this provides for policy direction remain valid and offer a roadmap for the skill structure transition needed to achieve economic prosperity.

## Supplementary Material

Appendix 01 (PDF)

## Data Availability

The replication files and city-level data supporting the findings of this study have been deposited in the Harvard Dataverse, available at https://doi.org/10.7910/DVN/YJVIOQ (51).
