# Supplementary material for: China’s demographic dividend has moved from age-based labor supply to skill-based productivity
Source: Proc Natl Acad Sci U S A. 2026 Apr 8;123(15):e2532906123. doi: 10.1073/pnas.2532906123 (PMC13079942; doi:10.1073/pnas.2532906123)
Supplement: Supplementary file 1 — Appendix 01 (PDF) [file pnas.2532906123.sapp.pdf]

PNAS

Supporting Information for
China's demographic dividend has moved from age-based labor supply to skill-based productivity

Hengyu Gu[a1], Yingju Wu[a1], Guillaume Marois[bc], Wolfgang Lutz[bc], Tianlong Niu[a]

Hengyu Gu, Guillaume Marois
Email: hygu@nju.edu.cn; marois@iiasa.ac.at

This PDF file includes:

Supporting text
Figures S1 to S10
Tables S1 to S9
SI References

[1] H.G. and Y.W. contributed equally to this work.

Supporting Information Text

1. Data Sources and Sample Screening. The data foundation for this research integrates multiple official and authoritative sources to ensure accuracy and reliability:

- **City Macroeconomic and Social Data:** Sourced from the China City Statistical Yearbook (2001–2021) and individual municipal statistical yearbooks, these provide the necessary data on gross domestic product (GDP), fixed asset investment, and other city-level control variables.
- **National Population Structure Data:** Derived from the aggregated data of China's three national population censuses in 2000, 2010, and 2020, as well as the aggregated data from the two national 1% population sample surveys in 2005 and 2015. The selection of 2020 as the study endpoint serves two purposes. First, this endpoint establishes a clear pre-pandemic baseline and thereby avoids the confounding effects of the anomalous demographic and economic shocks caused by the COVID-19 pandemic. Second, the 2020 National Population Census represents the most recent and comprehensive dataset available at the required prefectural level for constructing our explanatory variables. This data forms the core basis for constructing the age support ratios (ASR) and task-based skill ratio (TSR).
- **Population Projection Data for China:** Sourced from the World Population Prospects 2024 database (https://population.un.org/wpp/downloads) published by the Population Division of the United Nations Department of Economic and Social Affairs (1), this is used for long-term trend projection analysis.

We encounter a specific data challenge when constructing the 2020 TSR index because the 2020 Chinese National Population Census data only provide 1-digit occupational classifications. To ensure the integrity of the time series, we adopt the following alternative solution:

- **Standard Procedure:** We manually collect and compile the number of workers in 2-digit occupational groups for each prefecture-level unit from the publicly available 2020 population census yearbooks of each province, autonomous region, and municipality. This serves as the basis for calculating the TSR for that year.
- **Special Estimation for Xinjiang:** As the 2020 population census yearbook for Xinjiang is also not publicly available, we conduct a specific estimation of its TSR to ensure its sample representativeness in the western region. The estimation protocol is as follows: (1) Data Collection. We collect the number of workers in 1-digit occupational groups for each prefecture-level unit in Xinjiang (including prefecture-level cities, autonomous prefectures, etc.) from the Tabulation on the 2020 China Population Census by County. (2) Calculation of 1-digit

Occupational Group TSR. We aggregate the high-skill and low-skill levels of the 2-digit level occupations calculated for 2020 to the 1-digit occupational group level by averaging. (3) Estimation of TSR for Xinjiang. We apply the skill composition ratios calculated in the second step to the 1-digit occupational group data for Xinjiang obtained in the first step, thereby estimating the TSR for each prefecture-level unit in Xinjiang.

Due to the lack of necessary 2-digit occupational category statistics in the census data for some sample units (mainly counties under direct provincial jurisdiction) to construct the TSR index, the part of the analysis involving TSR ultimately covers 336 study units (including municipalities, prefecture-level cities, and provincially-administered counties). To more accurately identify the impact of the core explanatory variables and reduce omitted variable bias, this study incorporates a series of city-level control variables into the econometric models, including: per capita capital investment, urban employment, per capita government expenditure, total road mileage, number of newly established enterprises, foreign direct investment, and the share of the tertiary sector in GDP (*SI* Appendix, Table S5). During data processing, we identify that some cities lack data for control variables in certain years of the study period. To ensure a balanced panel and the robustness of the regression results, we remove these cities with missing data. After this screening process, the final sample for the empirical analysis consists of 289 cities. This sample exhibits high geographic representativeness, comprising cities distributed across China's Eastern, Central, Western, and Northeastern regions. It encompasses a diverse spectrum of cities at varying stages of economic development, providing a robust data foundation for the empirical analysis.

2. Methodology for Constructing the Task-Based Skill Ratio Index. This section details the multi-step methodology designed to construct the TSR index for China. Although China utilizes the National Occupational Classification Code (NOCC) to categorize workforce distribution, this system functions primarily as a registry of job titles. It lacks a granular, task-level repository comparable to the Occupational Information Network (O*NET) that quantifies the specific skill requirements and cognitive demands intrinsic to each occupation. Direct measurement of skill content using domestic data is infeasible. To overcome this data limitation, our framework infers skill attributes from the O*NET, the comprehensive U.S. database of occupational information, relying on the premise of industrial isomorphism. The process involves three primary stages: (1) quantifying a skill profile for each occupation based on O*NET's "Work Activities" data; (2) developing a robust protocol using Large Language Models (LLMs) to map Chinese occupational codes to their O*NET counterparts; (3) aggregating these occupational skill scores to the city level using employment data as weights.

2.1 An Introduction to the O*NET Database. The O*NET, sponsored by the U.S. Department of Labor, is the United States' primary and authoritative source for occupational information. Its principal value lies in its "content model", which deconstructs jobs into detailed, measurable components, most critically for this study, the "Work Activities" module associated with each occupation. These activities are quantitatively rated on multiple scales, such as Importance and Level, providing a rigorous, data-driven foundation for analyzing the underlying task composition of occupations. Given the absence of a comparable public database in China, O*NET's robust framework provides the essential foundation for our cross-national adaptation, enabling the construction of the TSR index used in this research.

2.2 Classification of Work Activities. Unlike existing standards that classify labor into manual or cognitive activities based on skill, this study focuses on extracting the Work Activities module from the O*NET database to define a skill score for different occupations. Based on their attributes, work activities are classified into three categories: high-skill, low-skill, and neutral activities, with the specific items selected referencing the *SI* Appendix, Table S6. The criteria for this classification are as follows:

- **Selection Criteria for High-Skill Activities:** Emphasis on information processing, creative thinking, strategic planning, and guiding personnel.
- **Selection Criteria for Low-Skill Activities:** Emphasis on machine operation, physical labor, and basic record-keeping.
- **Selection Criteria for Neutral Activities:** Significant variation in the content of the work activity across different occupations.

The TSR in this study is designed to quantify the propensity of O*NET occupations toward the high- and low-skill ends of the spectrum. Specifically, we select high- and low-skill work activities based

on our prior manual classification. Subsequently, using the "Level" score provided for each activity in the O*NET database, we calculate the average Level score for each occupation across the set of high-skill activities and the set of low-skill activities. These serve as the core metrics for an occupation's high- and low-skill levels.

Notably, neutral activities (e.g., Getting Information, Interacting with Computers) are not included in the above calculations. The primary reason is that the skill content and importance of such tasks exhibit immense heterogeneity across different occupations (for example, the requirements for "Getting Information" are vastly different for a journalist versus a factory worker). Including them in a unified quantitative framework would dilute the contrast between high and low skills and introduce measurement bias. Therefore, excluding neutral activities during the index construction is a critical step to ensure the final metric is robust and interpretable in cross-occupational and cross-city comparisons.

2.3 Occupational Concordance: Matching China's National Occupational Classification Code (NOCC) and the U.S. O*NET Standard Occupational Classification (SOC) System. Accurately applying the O*NET-based skill classification framework to the Chinese context requires establishing a reliable concordance mapping between occupational systems, which is a key methodological step in this study. Our goal is to match occupations from the 2009 edition of the NOCC of the People's Republic of China with the U.S. O*NET-SOC occupational system. Traditional matching methods, such as using the International Standard Classification of Occupations (ISCO) as an intermediary bridge, often suffer from reduced accuracy due to semantic drift in classification systems and the accumulation of errors during secondary conversions. To circumvent these drawbacks, this study designs and implements a direct semantic matching protocol based on LLMs, with cross-validation using two independent models (2, 3). Our decision to use LLMs for direct semantic matching is also supported by emerging research in related fields. For instance, recent studies have demonstrated through rigorous psychometric methods that LLMs can generate assessment items comparable to "gold-standard" human-authored textbook questions in key parameters such as difficulty and discrimination (4, 5). This finding indicates that LLMs are reliable in handling complex tasks that require a high degree of domain-specific knowledge, thus supporting our decision to apply them to the specialized domain of occupational classification matching.

Our matching protocol aims to map 3-digit NOCC codes to the most appropriate O*NET-SOC codes. To ensure objectivity and replicability, we employ a dual-model cross-validation strategy. Specifically, we independently feed the official name and full job description of each Chinese occupation into both ChatGPT-5 and Gemini 2.5 Pro, instructing them to perform the matching with O*NET-SOC, and then assess the cross-model consistency of the results. Before the formal matching, we screen the NOCC occupation list, primarily removing three types of occupations that lack a direct counterpart in the O*NET civilian occupational framework:

- Posts with unique characteristics due to institutional or cultural differences (e.g., "Notary").
- All directly related military occupations (e.g., "Weapons Engineering Technician").
- Residual "other" categories that are broadly defined and lack specific task descriptions.

This screening process ensures the validity of the sample to be matched, resulting in a core sample of 347 3-digit NOCC occupations, covering 85% of China's major occupational categories. To overcome the issue of endogenous skill changes that may occur within occupations over the two-decade study period, our matching process utilizes multiple versions of O*NET, from 5.0 to 27.2, and performs time-point optimal matching based on the official skill testing dates provided by O*NET, aiming for a more authentic and effective reflection of the high- and low-skill levels of different occupations at different times.

After obtaining skill scores at the 3-digit occupational level, we further perform an upward aggregation to the 2-digit occupational codes. This step is based on three main considerations: (1) Data Availability. The key census and population sample survey data used in this study are only available at the 2-digit occupational code level; (2) Time-Series Consistency. The 2015 revision of the NOCC consolidated some older occupational categories, leading to discrepancies between versions; (3) Mitigation of Mapping Errors. The correspondence between the NOCC and the O*NET SOC is not strictly one-to-one due to inherent differences in occupational definitions. Direct matching at the granular 3-digit level may introduce measurement noise. Aggregating to the 2-digit level effectively smooths out these discrepancies, thereby enhancing the robustness of the TSR.

To construct a uniform classification that is comparable over a longitudinal time series, we adopt a "bottom-up" aggregation strategy. This strategy completes the matching and skill assignment at the most granular 3-digit level, then determines the final skill level of a new version's 2-digit occupation by calculating the average skill scores of all its constituent 3-digit occupations. For example, the skill score for the 2-digit occupation "Engineering Technicians" in the 2015 NOCC is derived by averaging the scores of its subordinate 3-digit occupations, such as "Mechanical Engineering Technician" and "Electronics Engineering Technician".

Through this series of rigorous, semantics-focused matching and aggregation processes designed to harmonize time-series differences, we construct a skill profile for 73 Chinese 2-digit occupations, containing both a high-skill and a low-skill score (*SI* Appendix, Fig S7). This dataset not only reveals an expected hierarchy of skill intensity across the entire occupational landscape but also demonstrates significant heterogeneity in the internal skill structures of different jobs. The core advantage of this method is its ability to identify "intra-occupational skill polarity", meaning the gap between the high- and low-skill scores of different occupations is effectively recognized. We observe that managerial,

professional, and technical occupations (e.g., "Scientific Researcher", "Engineering Technician") generally exhibit significant skill polarity. This large gap indicates that these positions cover a wide range of responsibilities, from relatively routine administrative or technical functions to unstructured, complex problem-solving and strategic decision-making. In contrast, occupations concentrated in production, manufacturing, and related fields (e.g., "Textile Dyeing and Finishing Worker", "Tobacco Production and Processing Worker") show markedly narrower skill polarity. This compression of the skill range suggests that these occupations have a more homogenous and routinized task structure. It provides a more nuanced, structurally detailed data foundation for modeling and analyzing the complex dynamics of TSR in China's rapidly evolving economy.

2.4 Construction of the City-Level TSR. After completing the skill classification of work activities and the cross-national matching between the Chinese occupational classification and O*NET-SOC, the next step of this research is to aggregate the occupational-level skill measurements upward to construct a city-level TSR index. Drawing on the theoretical perspective that "a city can be viewed as a container of jobs, viewed as bundles of tasks and skills", we use the employment distribution across different occupations in each city as weights to integrate occupational skill characteristics into an overall skill profile for the city.

We calculate the city's overall high-skill level ($HighSkill_{ct}$) and low-skill level ($LowSkill_{ct}$). The specific method is to take a weighted average of each occupation's skill score and its employment share in that city. Let the high- and low-skill levels for occupation j be h_j and l_j, the employment share of occupation j in city c at time t be E_{jct}, and the total number of 2-digit occupations be J. This weighted averaging process ensures that dominant occupations with higher employment shares have a greater influence in shaping the city's TSR. The specific equation is:

$$HighSkill_{ct} = \sum_{j=1}^{J} (E_{jct} \times h_j)$$
$$LowSkill_{ct} = \sum_{j=1}^{J} (E_{jct} \times l_j) \qquad S(1)$$

To characterize the city's relative propensity toward the high- and low-skill ends of the spectrum, rather than the absolute scale of its skill stock, we define the TSR_{ct} as the ratio of the city's high-skill level to its low-skill level:

$$TSR_{ct} = \frac{HighSkill_{ct}}{LowSkill_{ct}} \qquad S(2)$$

TSR_{ct} reflects the compositional characteristics of the city's labor market. A higher TSR_{ct} indicates that the city's employment structure is more biased toward high-skill-intensive occupations; conversely, a lower value suggests that the city's labor structure is more reliant on low-skill-intensive

jobs. As this index is inherently a relative ratio rather than an absolute level, it is particularly well-suited for comparative analysis across cities and over time. Through the above process, we ultimately obtain a quantified TSR with a clear meaning. This index not only provides the core basis for the subsequent analysis of the skill landscape but also lays the data foundation for comparison with the results of the LLMs matching validation.

2.5 Robustness Checks. To verify the robustness and prudence of this manual classification framework, we introduce two leading LLMs (ChatGPT-5 and Gemini 2.5 Pro) for cross-comparative validation. We provide the models with a uniform structured prompt, asking them to perform the same three-category classification task for 40 activities, thereby enhancing the reliability of the research conclusions. The specific structured prompt is:

*"Please classify the following O*NET work activities into high-skill, low-skill, or neutral tasks, and for each activity provide the most likely category, the probability distribution across all three categories (percentages summing to 100%), and a concise justification for the classification, such as the task's core attributes, occupational applicability, or cognitive/physical complexity."*

The classification of the skill spectrum under different models reaches a high degree of consensus at both ends (*SI* Appendix, Table S7). For clearly defined core cognitive activities (e.g., "Analyzing Data or Information") and typical physical activities (e.g., "Performing General Physical Activities"), the results of the manual classification and both models are in complete agreement, which demonstrates the reliability of the classification framework in defining the two poles of the skill spectrum. However, the core difference in the validation lies in the handling of the neutral category. In the manual scheme, 60% of activities are classified as neutral to address the broad definitions in O*NET. The Gemini 2.5 Pro model largely recognizes the neutral category (with a 72.5% consistency with the manual classification), as its judgment logic is better able to identify the context-dependency of tasks. In contrast, the ChatGPT-5 model tends to "force-resolve" this ambiguity (50% consistency), leading to a classification somewhat disconnected from the complexity of occupational reality.

The quantified TSR indices further confirm this finding (*SI* Appendix, Fig S8). Despite differences in the underlying logic of the different classification methods, the measurement results from all three methods show a strong positive linear correlation ($R \geq 0.97$) between 2000 and 2020, demonstrating the overall robustness of using LLMs as a proxy for manual classification (*SI* Appendix, Fig S8, *A-J*). Furthermore, a comparison of core cities reveals a systematic difference: the average score curve from the Gemini model coincides with that of the manual classification, whereas the curve from ChatGPT-5 shows a significant and consistent upward deviation (*SI* Appendix, Fig S8, *K*). Moreover, robustness checks utilizing O*NET occupational skill levels fixed at the 2000, 2010, and 2020 benchmarks yield

results highly consistent with the baseline. This consistency effectively rules out potential biases arising from the selection of specific temporal benchmarks for skill measurement.

To further validate the conclusions from an economic effects perspective, we separately incorporate the TSR indices measured by the different paradigms into the baseline regression model. The results show that the promotional effect of all versions of the TSR index on economic growth is consistently positive and statistically significant, again confirming the reliability of this study's core findings (*SI* Appendix, Table S8).

In summary, the core conclusion of this cross-validation is that while all classification methods are highly consistent in their linear trends, they exhibit significant differences in how they handle ambiguous, context-dependent tasks. This difference serves as a reverse validation of the prudence of the manual classification scheme, which systematically uses the neutral category to handle broadly defined activities, thereby avoiding the introduction of subjective bias. At the same time, the results also indicate that Gemini 2.5 Pro, not only in trend but also in the fidelity of absolute values, is a more accurate automated replication of the manual classification judgment framework in this study.

3. Scenario-Based Projection of the TSR Compensation Effect: A Methodological Framework. This section constructs a dynamic projection framework to assess the required magnitude of improvement in China's TSR to offset the adverse impacts of population aging on economic growth under various demographic scenarios. The objective is to calculate the TSR level needed to neutralize the negative effects of a deteriorating ASR under the condition of a continuously increasing future demographic burden.

3.1 Data and Parameter Settings. To ensure the validity and cross-sample consistency of the projection, we make prudent settings for the data and parameters:

- **Population Projection Data:** Given China's latest official fertility statistics (6), the realization of scenarios other than the Low Fertility Variant (e.g., High, Medium, or Instant Replacement) is considered highly improbable. Therefore, this projection is rigorously grounded exclusively in the Low Fertility Variant (2025-2100) from the United Nations' World Population Prospects 2024 Revision as the sole realistic baseline. We extract the annual population counts by age cohort under this scenario to calculate the ASR directly (*SI* Appendix, Table S4).
- **Historical Parameter Scale:** To ensure that the future projection data are consistent with the data used in the regression model, all future ASR indicators are standardized using the parameters from this study's historical sample (2000–2020). Anchoring future data to the historical distribution is a critical step for ensuring the consistency of out-of-sample predictions. The specific "historical scale" parameters are detailed in *SI* Appendix, Table S9.
- **Baseline Year Setting:** The projection sets 2020 as the baseline year. This choice is not only because it is the last observation point in our panel data, but also because it represents a stable, referenceable level of the combined contribution of demographic factors to the economy. The skill requirements in all future scenarios are aimed at maintaining this baseline level. The actual observed parameters for 2020 are detailed in *SI* Appendix, Table S9.
- **Source of Regression Parameters:** The core parameters required for the projection are derived from the estimation results of the baseline regression model (Eq. 6), respectively capturing the marginal contributions of the ASR, TSR, and their interaction to GDP per capita.

3.2 Definition of Working-Age Boundaries and Dynamic Coefficient Evolution. To comprehensively assess the compensatory effect of the TSR, this study constructs a dual dynamic projection framework that relaxes static assumptions by varying both working-age definitions and economic structural parameters.

Regarding the demographic dimension, we define the baseline working-age population as those aged 15-64. Furthermore, referencing Rees, *et al.* (7), we extend the upper age limit to 69 (implying retirement at 70) and 74 (implying retirement at 75) in alternative scenarios. The primary objective of these settings is to quantify the TSR compensation level required to neutralize the adverse shocks of population aging under varying statistical definitions of the potential labor force.

Regarding the structural parameters, the coefficients derived from the estimation of Eq. 6 using panel data from 289 cities (2000-2020) exhibit robust statistical significance. As a synthesis of historical economic patterns, these parameters possess a degree of external validity for extrapolation. However, acknowledging the potential long-term impact of Skill-Biased Technological Change on productivity, we introduce two dynamic scenarios alongside the baseline static scenario ($\Delta\beta = 0$) to examine the model's sensitivity to structural evolution rigorously:

- **Age-Dynamic Scenario:** Assuming the marginal contribution coefficient of the ASR (β_1) grows at an annual rate of 1%;
- **Skill-Dynamic Scenario:** Assuming the marginal contribution coefficient of the TSR (β_2) grows at an annual rate of 1%.

It is important to emphasize that these dynamic scenarios are designed as a stress test or sensitivity analysis, rather than a precise forecast of economic parameters for specific future years. These assumptions allow us to evaluate the effective boundaries of the TSR compensation mechanism contingent on specific structural evolutions, thereby providing a more robust and flexible reference range for policy formulation. Because the inherent complexity of long-term economic forecasting precludes precise empirical prediction, we introduce a stylized constant growth rate to simulate anticipated structural shifts:

Regarding the Age-Dynamic Scenario (β_1), our assumption is rooted in the theory of factor scarcity. Although historical data may suggest a fluctuating or milder marginal impact of aging, the projection period (2025-2100) involves a drastic contraction of the labor force. As labor becomes a binding constraint on growth, the marginal output loss associated with each unit of labor decline is theoretically expected to rise. Setting a 1% annual growth in β_1 captures this intensifying structural penalty driven by increasing factor scarcity, reflecting the constraints on economic growth inherent in the deep aging phase.

Regarding the Skill-Dynamic Scenario (β_2), our assumption reflects the trajectory of technological evolution. Given the ongoing technological change characterized by artificial intelligence and digitization, skill-biased technological change is expected to amplify the returns to human capital. We calibrate this growth at a stylized benchmark of 1% per annum. This parameterization serves as a prudent

heuristic, capturing the structural tendency of rising skill premiums while avoiding the risk of implausible exponential divergence associated with linear extrapolation of historical high-growth phases.

3.3 Derivation of the Dynamic Compensation Equation. Our core task is to solve for the required TSR level ($TSR^*_{t,comp}$) in a future year *t* that can fully compensate for the negative impact of the ASR in that year (ASR^*_t). The principle is that the combined economic effect determined by the ASR and TSR in the future should remain consistent with the baseline effect in 2020 to achieve full compensation for the rising demographic burden. The equation is:

$$\beta_1 ASR^*_t + \beta_2 TSR^*_{t,comp} + \beta_3 (ASR^*_t \times TSR^*_{t,comp}) = \beta_1 ASR^*_{2020} + \beta_2 TSR^*_{2020} + \beta_3 (ASR^*_{2020} \times TSR^*_{2020}) \qquad S(3)$$

Expanding Eq. S(3), we get:

$$(\beta_2 + \beta_3 ASR^*_t) TSR^*_{t,comp} = C - \beta_1 ASR^*_t \qquad S(4)$$

where $C = \beta_1 ASR^*_{2020} + \beta_2 TSR^*_{2020} + \beta_3 (ASR^*_{2020} \times TSR^*_{2020})$ is a constant calculated from the 2020 data and regression coefficients. By solving algebraically, we obtain the final dynamic compensation equation:

$$TSR^*_{t,comp} = \frac{C - \beta_1 ASR^*_t}{\beta_2 + \beta_3 ASR^*_t} \qquad S(5)$$

where $TSR^*_{t,comp}$ represents the standardized TSR required in a future year t to offset the adverse impact of the ASR in that year, i.e., the "compensatory" TSR demanded by the demographic changes.

3.4 Handling Model Instability: The Denominator Floor. When directly applying Eq. S(5), we find that the model has a critical threshold: when the future ASR deteriorates to an extreme level, the denominator ($\beta_2 + \beta_3 ASR^*_t$, i.e., the marginal rate of return to skills) can approach zero or even become negative. This phenomenon is a statistical artifact that can occur when a linear model is extrapolated to bounds far from the data's central tendency. It leads to mathematical instability (the result approaches infinity), and its economic interpretation requires particular caution. To resolve this issue and ensure the economic meaningfulness and robustness of the projection results throughout the entire forecast horizon, we introduce a methodologically prudent assumption: the marginal rate of return to skills has an effectiveness floor. We posit that even in the worst demographic context, the economic effect of improving the TSR will not completely disappear or turn negative but will remain at a minimum positive level. Specifically, we set a floor value for the denominator. This choice represents a relatively conservative assumption about the bottom line of returns to TSR. The revised denominator is calculated as follows:

$$Denominator_t = max(\beta_2 + \beta_3 ASR^*_t, \text{floor}) \qquad S(6)$$

where the denominator term ($\beta_2 + \beta_3 ASR^*_t$) represents the age-adjusted marginal contribution of the TSR to GDP per capita, which is subject to a technical lower bound constraint (floor, ϵ) to ensure numerical stability. In the baseline projection, we set this floor at $\epsilon = 0.01$, a conservative threshold

given that the empirically estimated main effect of skills ($\beta_2 \approx 0.09$) ensures the constraint remains non-binding for the vast majority of the projection period; sensitivity tests further confirm this robustness, showing that a minimal constraint ($\epsilon = 0.001$) yields trajectories identical to the benchmark, while a strict constraint ($\epsilon = 0.05$) causes divergence only after 2060 with a maximum deviation of approximately 20% (*SI* Appendix, Fig S9), demonstrating that the structural validity of the projection results remains robust and is not fundamentally altered by the specific calibration of the technical lower bound.

3.5 Inverse Standardization and Policy Metric Restoration. The compensatory TSR ($TSR^*_{t,comp}$) derived from Eq. S5 represents a standardized index (Z-score) relative to the historical distribution. To translate this statistical abstraction into a tangible policy metric, we apply an inverse standardization procedure to restore the original scale of the TSR:

$$TSR_{t,real} = TSR^*_{t,comp} \times \sigma_{TSR} + \mu_{TSR} \qquad S(7)$$

where μ_{TSR} (mean) and σ_{TSR} (standard deviation) are the statistical parameters derived from the 2000–2020 historical sample (detailed in *SI* Appendix, Table S9). Anchoring the transformation to historical parameters is methodologically critical. It ensures that the projected skill requirements are not arbitrary theoretical values but are consistent with China's historical technological-skill trajectory. The resulting $TSR_{t,real}$ represents the TSR required in year t, providing a direct, interpretable benchmark for education and labor market policymaking.

Figures

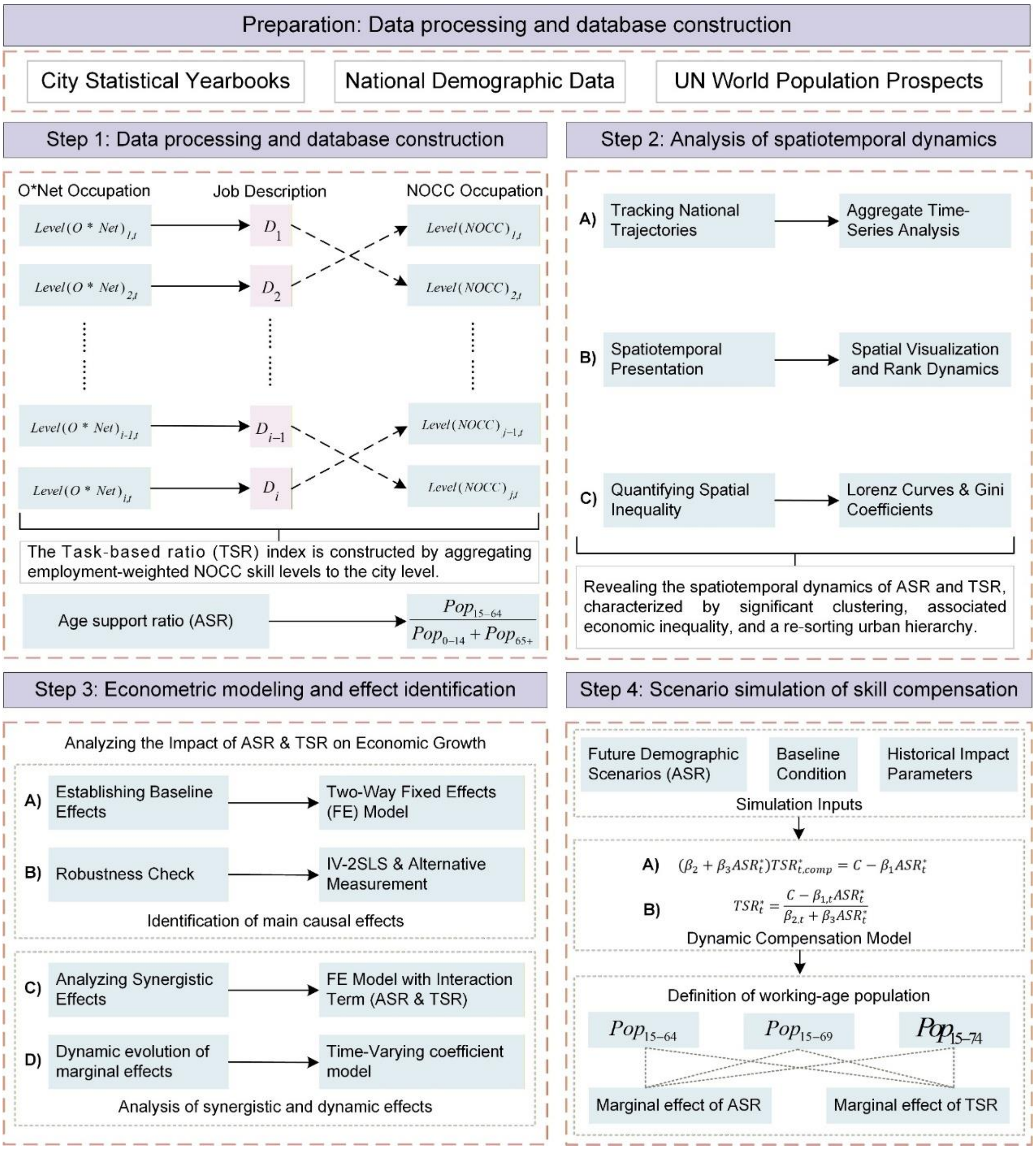

Fig. S1. Schematic of the research framework. Step 1 details the variable construction process, where $Level(O^{*}Net)_{i,t}$ denotes the skill score of the i_{th} U.S. occupation derived from the t-th version of the O*NET database. This is used, via semantic matching of the job description (D_j), to derive $Level(NOCC)_{j,t}$, the corresponding skill score for the j_{th} Chinese occupation. Subsequent steps then outline the analysis of these derived variables (Step 2), the econometric strategy to identify their economic impacts (Step 3), and the projection of future scenarios (Step 4).

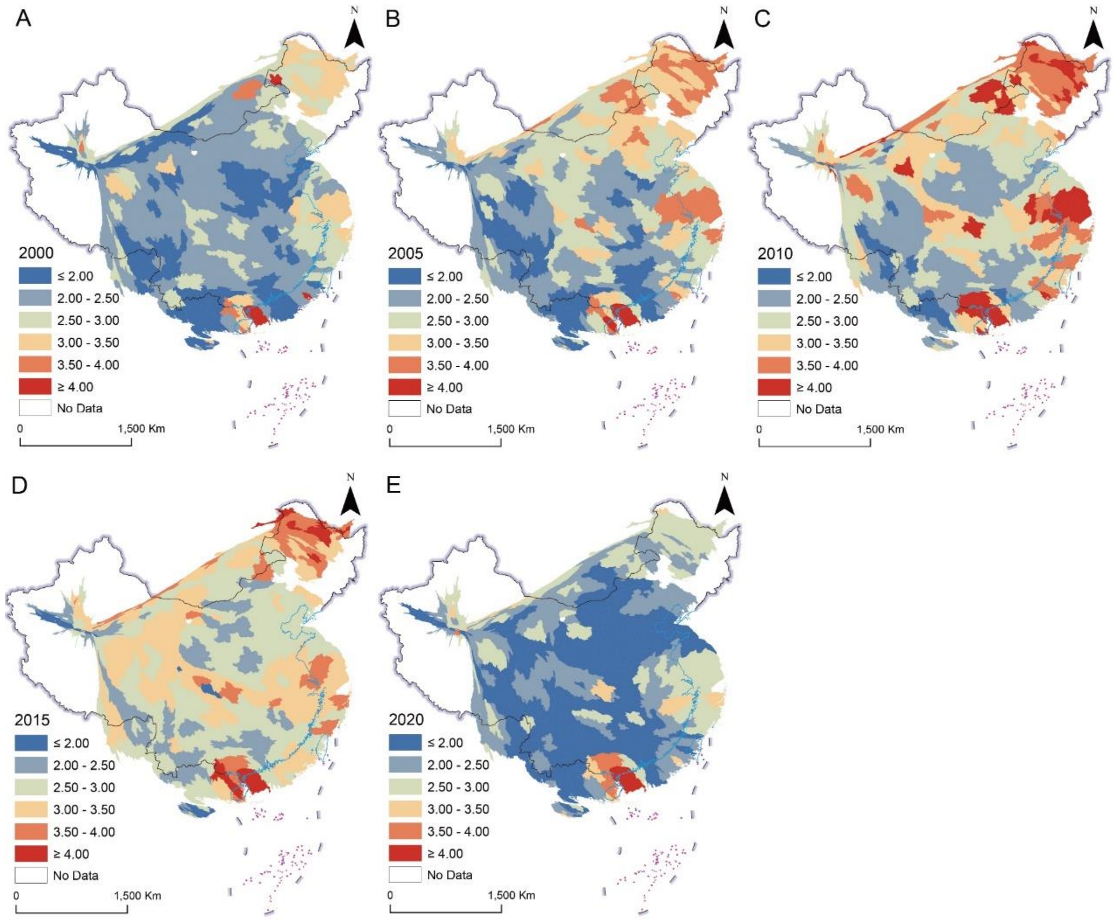

Fig. S2. Spatial distribution of the ASR weighted by population size. This figure displays the spatial patterns of the ASR across Chinese cities from 2000 to 2020 (A-E) using population cartograms. To avoid visual bias in large but sparsely populated administrative areas, all cities are scaled proportionally to their total population using the diffusion-based algorithm developed by Gastner and Newman (8). This approach ensures that the research findings are visually centered on major population hubs rather than the entire administrative landmass.

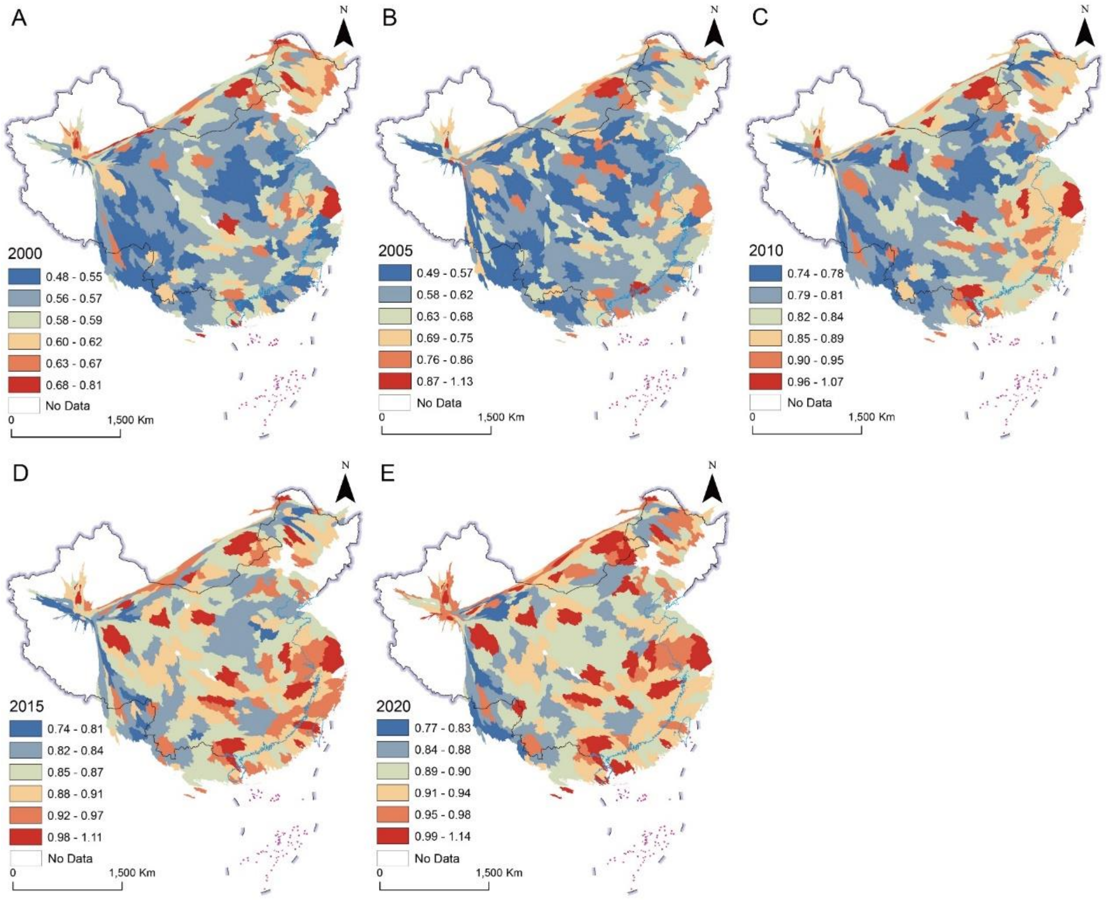

Fig. S3. Spatial distribution of the TSR weighted by population size. (A-E) display the spatial patterns of the TSR across Chinese cities from 2000 to 2020. The cartographic method and population-weighting algorithm are consistent with those described in *SI* Appendix, Fig S2.

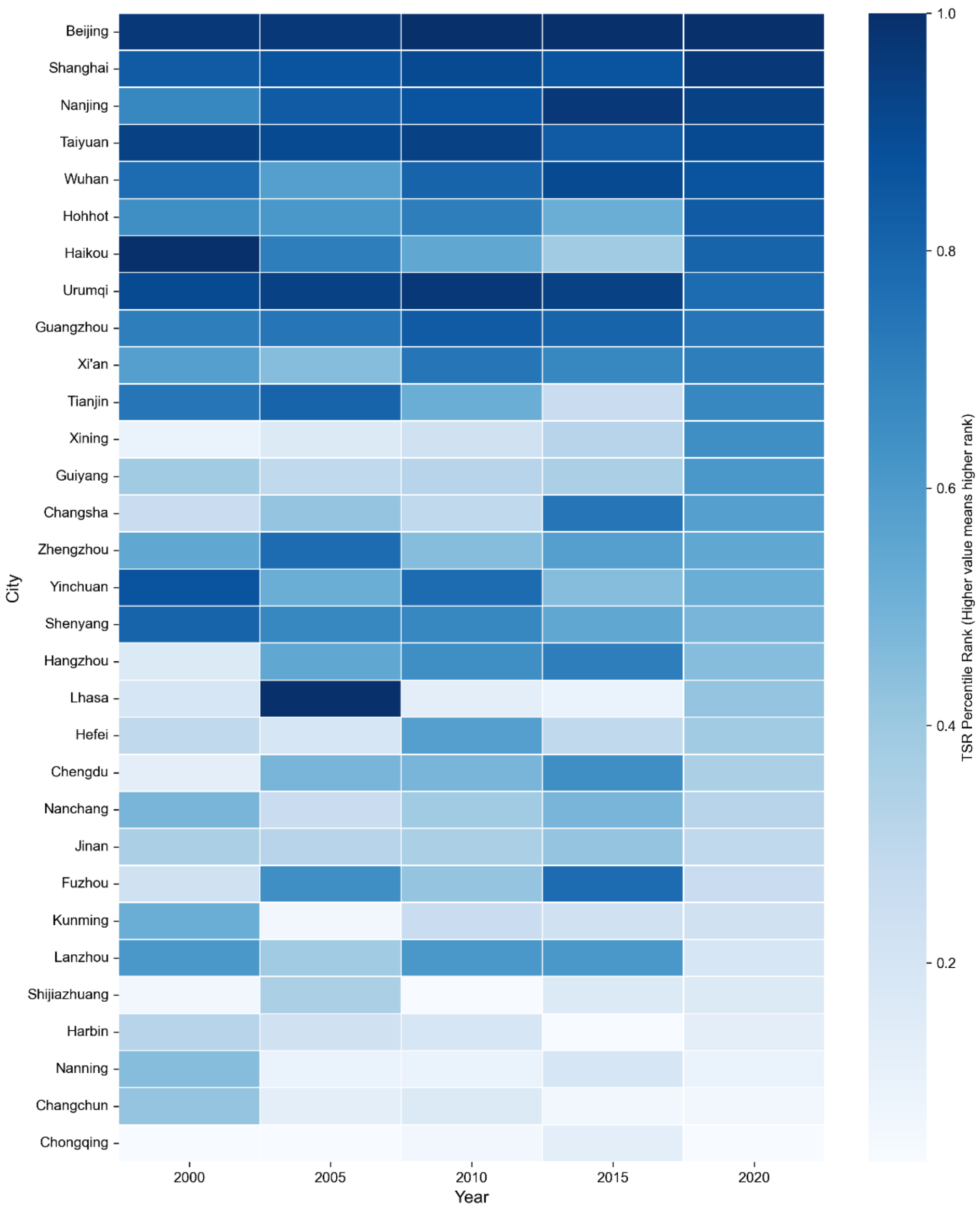

Fig. S4. Change in TSR rankings for major Chinese cities, 2000–2020. This figure illustrates the change in TSR rankings for 31 of China's provincial capitals and municipalities from 2000 to 2020. Each cell's value and color represent the percentile rank of a city's TSR score relative to the other cities in the sample for that specific year. A higher value (darker color) indicates a higher relative rank. Cities on the y-axis are sorted in descending order based on their 2020 ranking to highlight the current hierarchy.

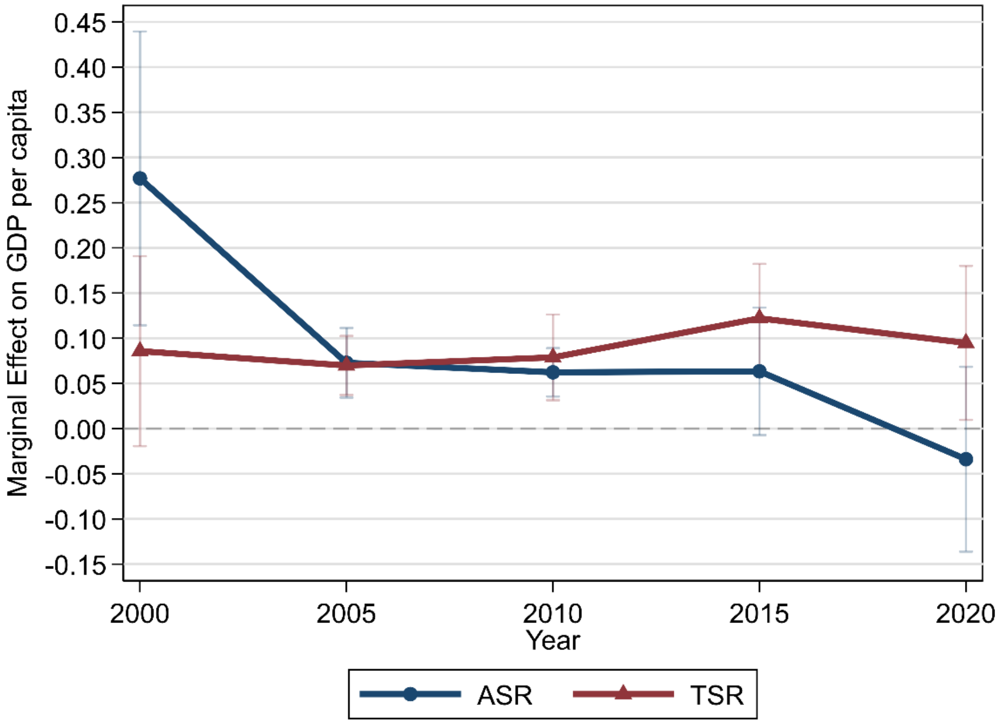

Fig. S5. Dynamic evolution of the marginal effects of industrial structure on economic growth, 2000–2020. This figure illustrates the time-varying impacts of ASR and TSR on economic development. To capture these temporal dynamics, the baseline specification is extended by interacting the key explanatory variables and their interaction term with year dummy variables (D_t), modeled as: $\ln(\text{GDP per capita}_{ct}) = \alpha + \sum_{t=2000}^{2020} \beta_{1t}(\text{ASR}_{ct} \times D_t) + \sum_{t=2000}^{2020} \beta_{2t}(\text{TSR}_{ct} \times D_t) + \sum_{t=2000}^{2020} \beta_{3t}(\text{ASR}_{ct} \times \text{TSR}_{ct} \times D_t) + \gamma \mathbf{X}_{ct} + \mu_c + \varepsilon_{ct}$. The vertical bars indicate 95% confidence intervals clustered at the city level. The horizontal dashed grey line at zero represents the threshold of statistical significance; estimates with intervals strictly above or below this line indicate that the structural factor significantly influenced economic growth during that specific year. This pattern illustrates the shifting dominance of structural drivers over the two decades.

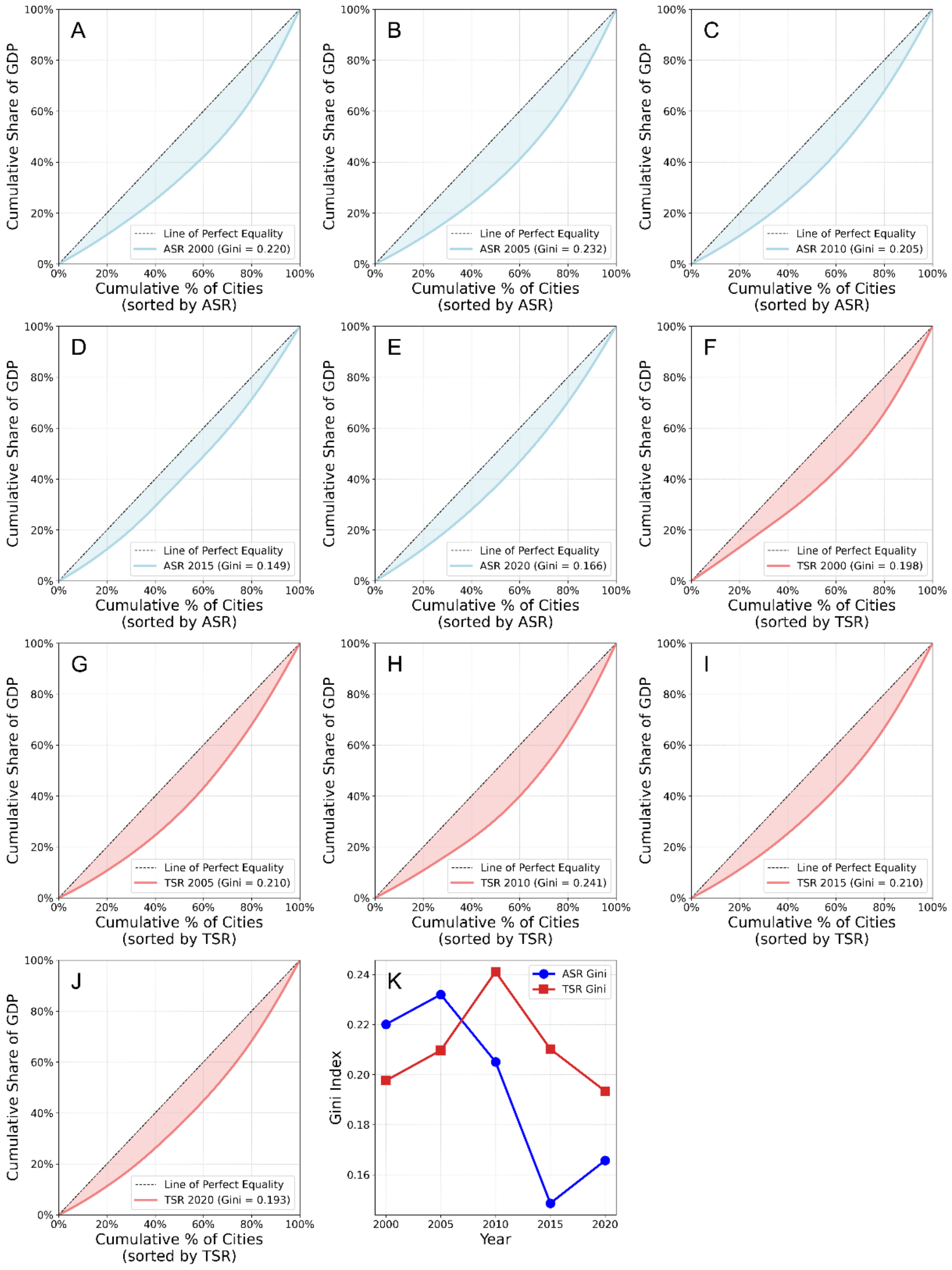

Fig. S6. ASR and TSR as drivers of China's spatial economic inequality. The figure quantifies the spatial inequality of GDP from 2000 to 2020 using Lorenz curves and Gini coefficients. (A-E) and (F-J) show inequality based on city rankings by ASR and TSR, respectively. (K) plots the Gini coefficients over time.

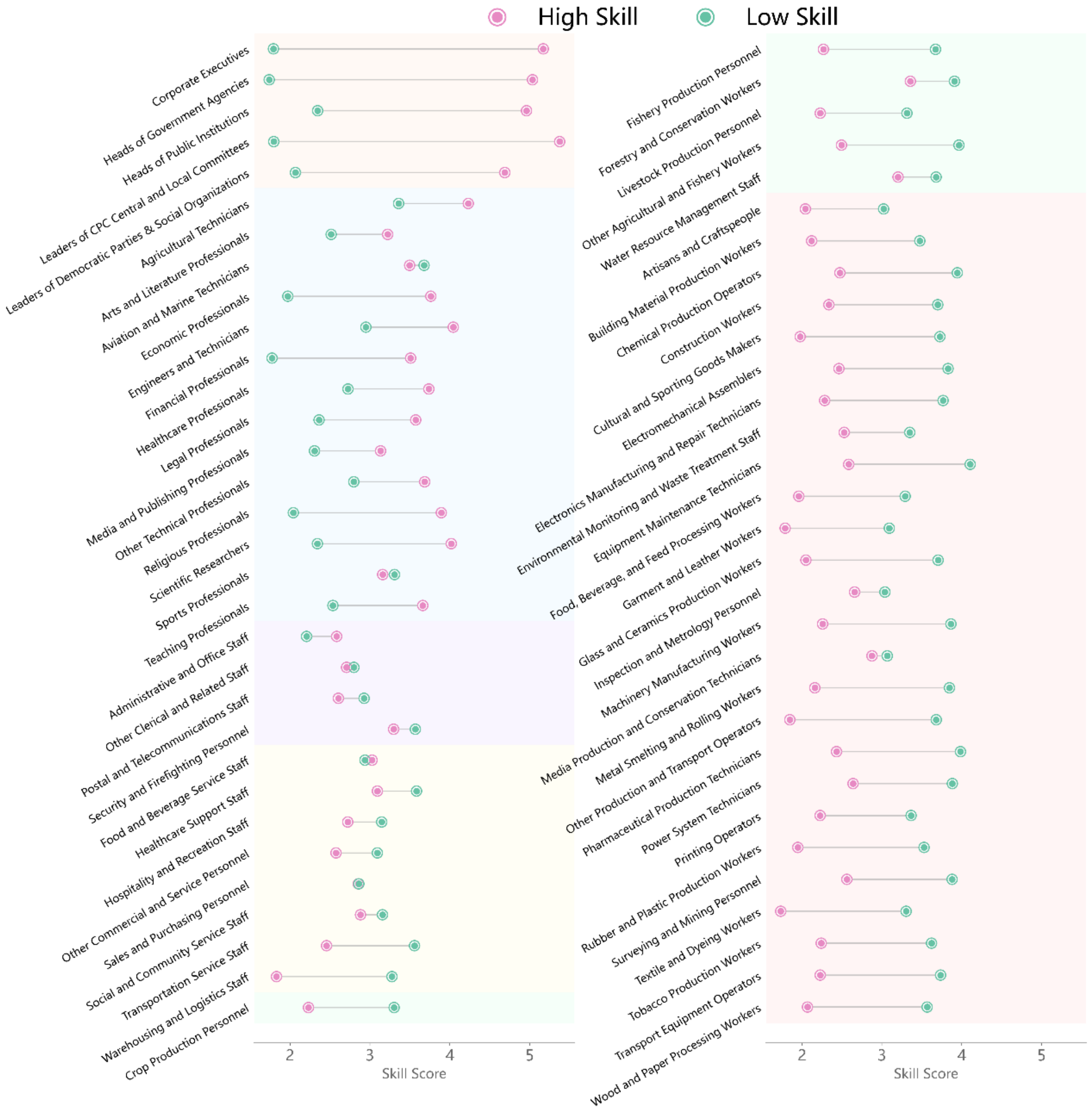

Fig. S7. Quantification of skill levels for Chinese occupations. This figure illustrates the high- and low-skill levels for all 73 2-digit Chinese occupations. The high-skill and low-skill scores for each occupation are derived by establishing a semantic match between China's NOCC and the U.S. O*NET database. Occupations are grouped by their major categories (1-digit occupations), which are distinguished by different background colors. Across all groups, the high-skill intensity scores average 2.91 (SD=0.89), while the low-skill intensity scores average 3.16 (SD=0.65), indicating substantial heterogeneity in task composition.

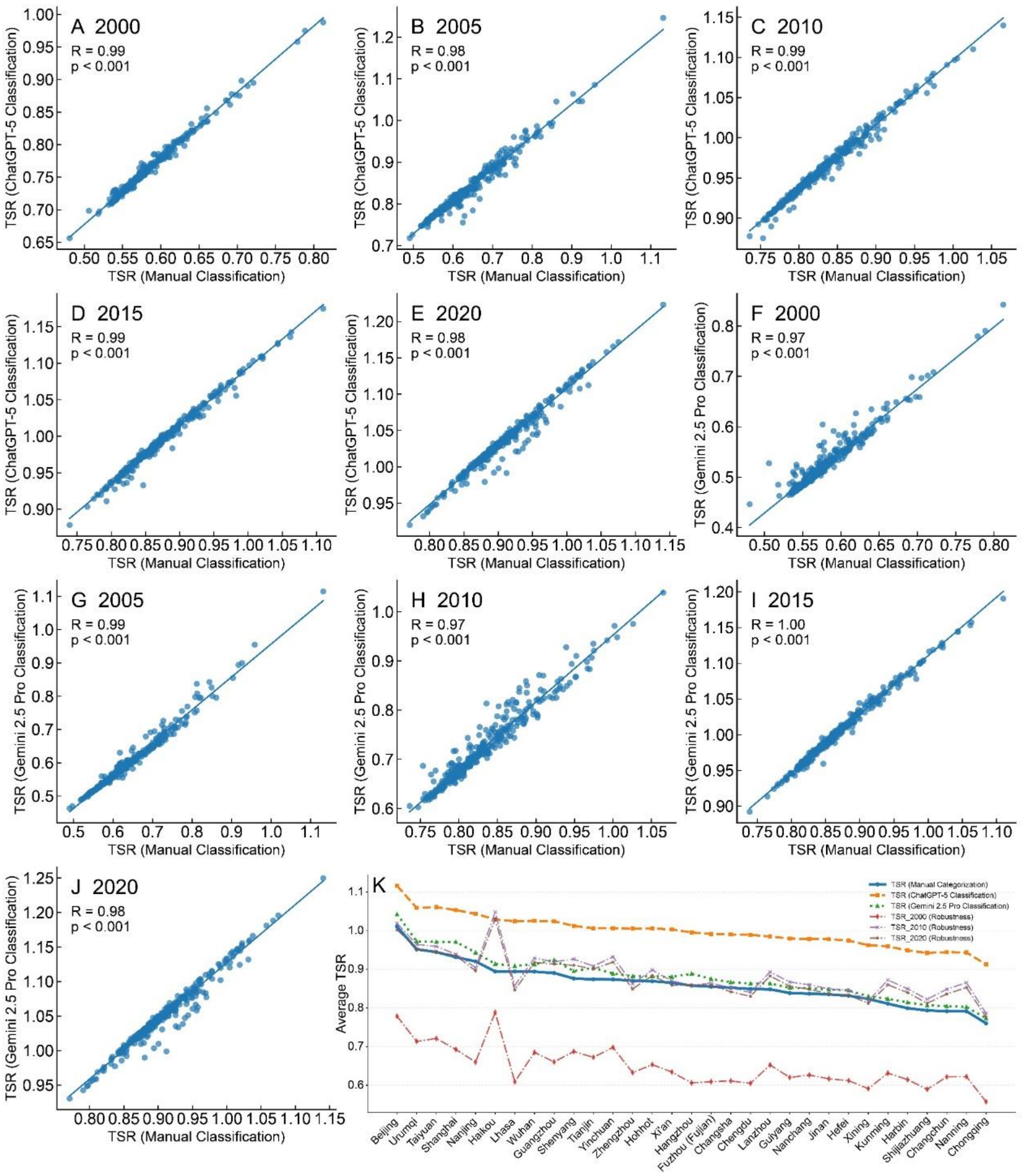

Fig. S8. High concordance between manual and LLM-based classifications of TSR. Validation of the TSR metrics. (A-J) Scatter plots correlate the manually categorized TSR metric with metrics derived from (A-E) ChatGPT-5 and (F-J) Gemini 2.5 Pro for the years 2000, 2005, 2010, 2015, and 2020. Each point represents a city, the solid line is the best-fit regression, and Pearson's R and p-values are indicated in each panel. (K) The five methods are compared across China's provincial capitals and municipalities, with cities sorted by the manual metric's average score. The plot reveals high concordance among the methods in both rank-order and magnitude.

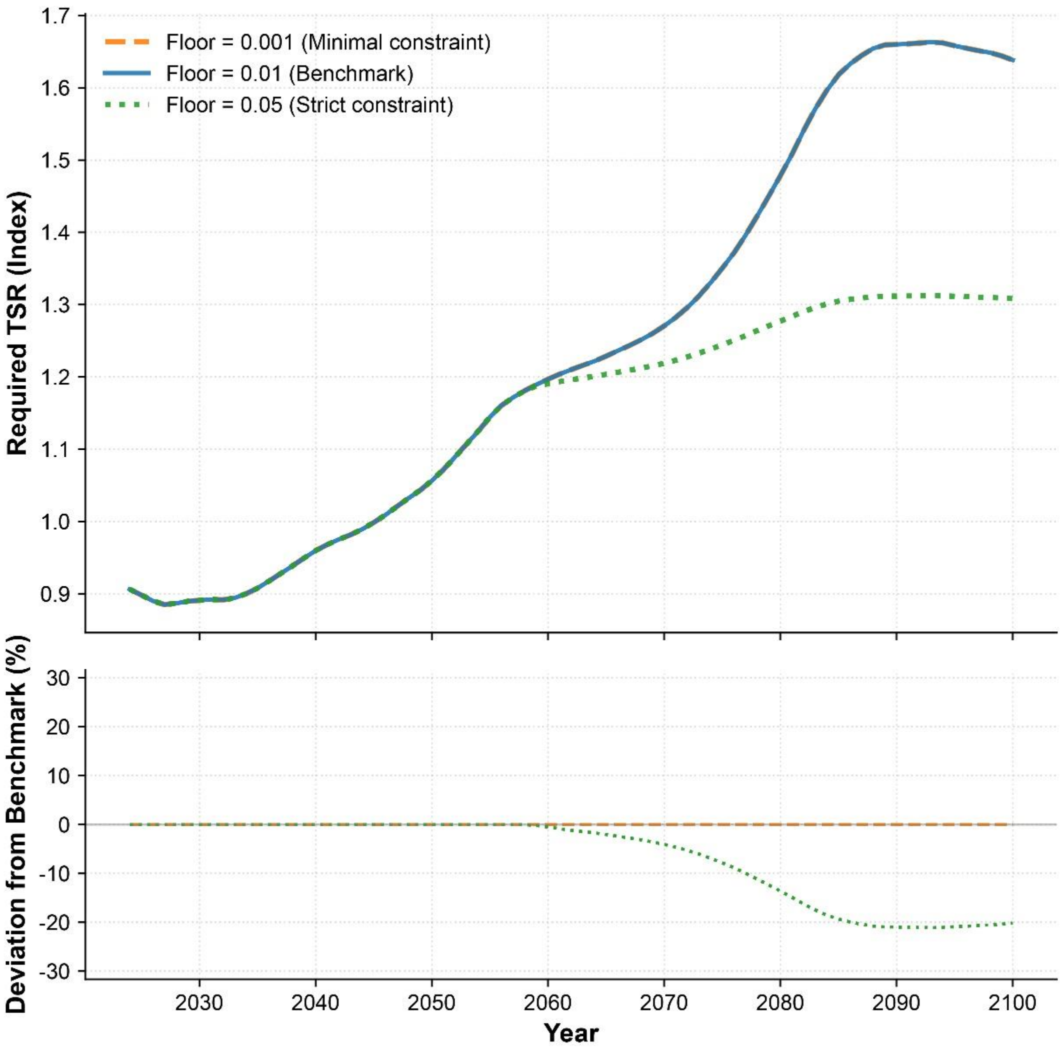

Fig. S9. Sensitivity analysis of the projected required TSR to the denominator floor parameter. The upper panel presents the projected trajectories of the TSR under the Baseline scenario (Retirement age 65 with static coefficients), calculated using three distinct floor thresholds: $\epsilon = 0.001$ (minimal constraint), $\epsilon = 0.01$ (benchmark), and $\epsilon = 0.05$ (strict constraint). The lower panel displays the percentage deviation of the alternative specifications ($\epsilon = 0.001$ and $\epsilon = 0.05$) relative to the benchmark ($\epsilon = 0.01$) over the projection period from 2020 to 2100.

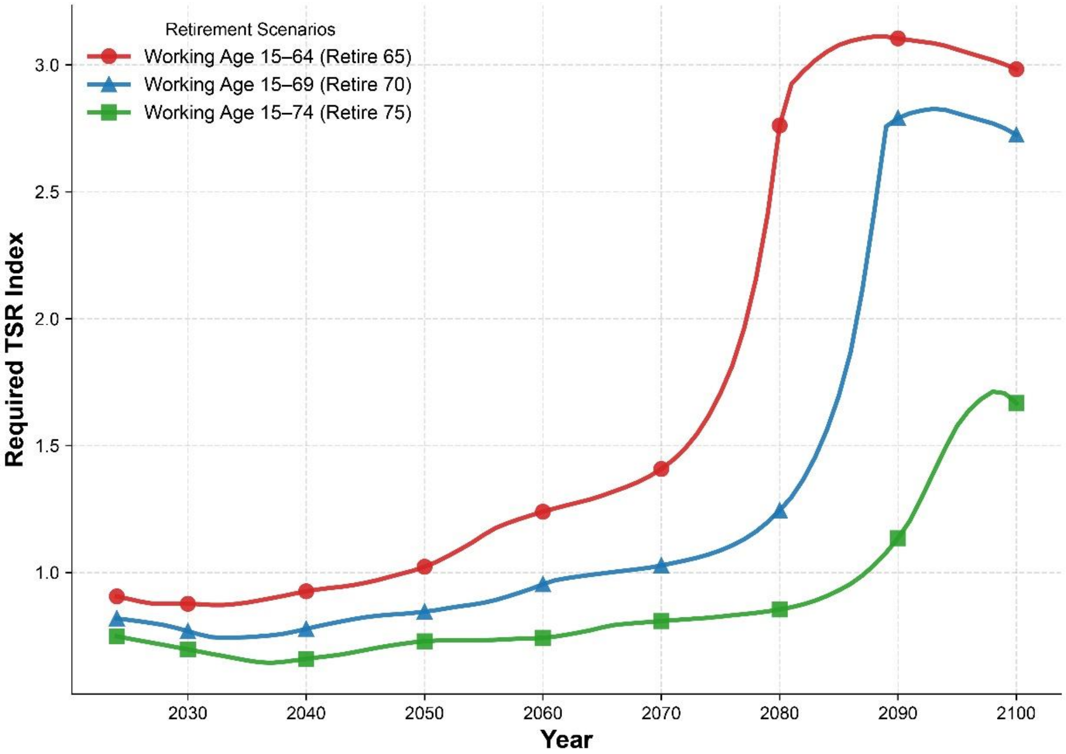

Fig. S10. Projections of the required TSR under dual dynamic pressure. The projections map the compensatory TSR trajectories (2025–2100) required to sustain economic momentum under a compound stress-test scenario. The analysis interacts three definitions of the working-age population (upper age limits at 65, 70, and 75 years) with a "Dual Dynamic" mechanism: a simultaneous annual 1% decay in the marginal return to TSR (β_2), reflecting diminishing returns or skill saturation, and an annual 1% exogenous growth in the ASR value, simulating demographic recovery or automation-induced substitution. An effectiveness floor ($\epsilon = 0.01$) is imposed on the denominator to prevent mathematical singularity under extreme parameter divergence.

Tables

Table S1. IV-2SLS regression results on the impact of ASR and TSR on economic development.

	(1)		(2)
	ASR	TSR	IV-2nd
IV-ASR	0.515***		
	(0.066)		
IV-TSR		0.550***	
		(0.025)	
ASR			0.061***
			(0.016)
TSR			0.211***
			(0.059)
Controls	Yes	Yes	Yes
Year fixed effects	Yes	Yes	Yes
City fixed effects	Yes	Yes	Yes
Sanderson-Windmeijer (SW) F-statistic	59.67	273.02	
Kleibergen-Paap rk LM statistic			76.575
Kleibergen-Paap rk Wald F statistic			27.114
Observations	1420	1420	1420

Note. Table S1 presents the IV-2SLS regression results. To address potential endogeneity, we instrument for ASR and TSR using the "leave-one-out" mean values from other cities within the same province(9). The four municipalities are excluded as this strategy is not applicable to single-city provincial units. The validity of the instruments is confirmed by diagnostic tests. The Kleibergen-Paap rk LM statistic rejects the null hypothesis of under-identification ($p < 0.01$). Furthermore, as the model includes two endogenous regressors, we report the Sanderson-Windmeijer (SW) F-statistic for each. The SW F-statistics for ASR and TSR are 59.69 and 271.79, respectively, both well above the conventional threshold of 10, thus ruling out weak instrument concerns.

Table S2. Impacts of ASR and TSR on economic development across different city sizes.

	Small & Medium-sized Cities		Large Cities	
	(1)	(2)	(3)	(4)
ASR	0.045***	0.051***	0.027**	0.026**
	(0.014)	(0.014)	(0.013)	(0.013)
TSR	0.051**	0.062***	0.155***	0.153***
	(0.022)	(0.022)	(0.031)	(0.032)
ASR×TSR		0.021**		0.006
		(0.010)		(0.008)
Ln Pcinvest	0.069***	0.074***	0.034	0.038*
	(0.020)	(0.020)	(0.023)	(0.022)
Ln Patent	0.014*	0.016**	0.026***	0.026***
	(0.008)	(0.008)	(0.010)	(0.010)
Ln Population	-0.851***	-0.853***	-0.822***	-0.832***
	(0.084)	(0.081)	(0.057)	(0.061)
Ln Road	-0.014	-0.009	0.008	0.010
	(0.027)	(0.027)	(0.018)	(0.018)
Ln Newfirms	0.022**	0.020*	0.020	0.020
	(0.011)	(0.011)	(0.017)	(0.017)
Ln Fdi	0.016***	0.016***	0.018***	0.018***
	(0.004)	(0.004)	(0.007)	(0.007)
Tertiaryshare	-0.004***	-0.004***	-0.001	-0.002
	(0.001)	(0.001)	(0.002)	(0.002)
Constant	12.582***	12.514***	13.700***	13.684***
	(0.560)	(0.547)	(0.560)	(0.550)
Observations	940	940	505	505
Year fixed effects	Yes	Yes	Yes	Yes
City fixed effects	Yes	Yes	Yes	Yes
R^2	0.980	0.980	0.985	0.985

Note. The classification of city sizes follows the Notice of the State Council on Adjusting the Criteria for City Size Classification (10). Based on the urban permanent population, cities are categorized into two groups: Large cities are defined as those with a population of 1 million or more (aggregating Type II large, Type I large, super-large, and megacities), while small & medium-sized cities comprise those with a population of less than 1 million.

Table S3. Impacts of ASR and TSR on economic development across migration patterns.

	Low Net Inflow		High Net Inflow	
	(1)	(2)	(3)	(4)
ASR	0.044*	0.049**	0.045***	0.044***
	(0.023)	(0.024)	(0.014)	(0.014)
TSR	0.061***	0.064***	0.085***	0.086***
	(0.016)	(0.016)	(0.031)	(0.031)
ASR×TSR		0.008		0.016**
		(0.011)		(0.008)
Ln Pcinvest	-0.027	-0.025	0.065***	0.071***
	(0.023)	(0.023)	(0.016)	(0.016)
Ln Patent	-0.003	-0.004	0.031***	0.031***
	(0.009)	(0.009)	(0.008)	(0.008)
Ln Population	-0.849***	-0.843***	-0.782***	-0.792***
	(0.075)	(0.072)	(0.068)	(0.068)
Ln Road	-0.014	-0.013	0.014	0.018
	(0.016)	(0.016)	(0.021)	(0.021)
Ln Newfirms	0.057***	0.059***	0.020*	0.019*
	(0.017)	(0.016)	(0.010)	(0.010)
Ln Fdi	0.011	0.011	0.014***	0.013**
	(0.007)	(0.007)	(0.005)	(0.005)
Tertiaryshare	0.000	0.000	-0.007***	-0.007***
	(0.001)	(0.001)	(0.001)	(0.001)
Constant	13.592***	13.524***	12.551***	12.495***
	(0.504)	(0.491)	(0.510)	(0.502)
Observations	497	497	948	948
Year fixed effects	Yes	Yes	Yes	Yes
City fixed effects	Yes	Yes	Yes	Yes
R^2	0.988	0.988	0.977	0.977

Note. The sample is divided into high net inflow and low net inflow groups based on the net migration location quotient (LQ). The LQ is calculated as $LQ_{ct} = ((P_{ct}^{res} - P_{ct}^{hukou})/P_{ct}^{res})/((\sum P_{ct}^{res} - \sum P_{ct}^{hukou})/\sum P_{ct}^{res})$, where P_{ct}^{res} and P_{ct}^{hukou} denote the resident and registered populations of city c in year t, respectively, and the denominator represents the national average net migration rate. High net inflow cities are defined as those with an LQ greater than the sample mean, while low net inflow cities comprise the remainder. This specification reflects the unique institutional context of China, where the household registration (*Hukou*) system governs access to public welfare. Obtaining a local *Hukou* typically requires overcoming substantial administrative thresholds (e.g., skill or property requirements) and grants full access to local social rights. Therefore, this indicator posits that migrants who acquire a local *Hukou* have completed their localization transition, distinguishing them from the institutionally floating population captured by the net inflow metric.

Table S4. Demographic characteristics under different working-age definitions

Definition Scenario	Working-Age Definition	Dependency Definition	Mean ASR
Baseline Definition	15–64	0–14 & 65+	1.254
Moderate Extension	15–69	0–14 & 70+	1.797
Deep Extension	15–74	0–14 & 75+	2.643

Note. Table S4 presents the average demographic characteristics from 2025 to 2100 based on the UN Low Variant projection. Instead of comparing different fertility scenarios, we focus on quantifying the compensatory magnitude of the TSR required under varying definitions of the working-age boundary (65, 70, and 75 years). These scenarios, based on the detailed methodology and assumptions from the United Nations, capture multiple possible future demographic trajectories and provide a critical basis for analyzing the impact of population age structure on economic development (1).

Table S5. Descriptive statistics of variables

Variables	Indicator	Obs.	Mean	S.D	Min	Max
GDP per capita	Ln (GDP per capita, constant price)	1445	9.796	0.890	7.832	11.556
ASR	$\frac{\text{Population}_{15-64}}{\text{Population}_{<15}+\text{Population}_{>64}}$(standardized)	1445	-0.023	0.856	-1.592	2.723
TSR	Task-based skill ratio index (standardized)	1445	-0.002	0.994	-1.584	1.813
Ln Pcinvest	$Ln\left(\frac{\text{Stock of Capital Investment}}{\text{Population}}\right)$	1445	13.256	1.058	10.633	15.284
Ln Patent	$Ln\ (Invention\ Patent)$	1445	3.440	2.279	0.000	9.122
Ln Population	$Ln\ (Poputation)$	1445	5.820	0.704	3.837	7.467
Ln Road	Ln (Total Road Mileage)	1445	8.877	0.843	6.441	10.396
Ln Newfirms	Ln (Number of New Firms)	1445	9.689	1.157	6.290	12.234
Ln Fdi	Ln (Foreign Direct Investment)	1445	4.508	2.149	0.004	8.925
Tertiaryshare	$\frac{\text{Tertiary Industry}}{\text{GDP}} \times 100$	1445	39.657	9.556	17.530	68.880

Note. Table S5 reports the descriptive statistics of the key variables used in the regression analyses. All variables are based on a balanced panel of 1,445 observations at the city level. To mitigate the influence of outliers and ensure the robustness of regression results, all continuous variables have been winsorized at the 1st and 99th percentiles. Both ASR and TSR have been standardized to have a mean of zero and a standard deviation of one, with TSR further constructed as described in Eq. S2 in the *SI* Appendix.

Table S6. Classification of O*NET work activities into high- and low-skill tasks

Category	Work Activities (from O*NET)
High-skill activities	Analyzing Data or Information
	Thinking Creatively
	Making Decisions and Solving Problems
	Developing Objectives and Strategies
	Updating and Using Relevant Knowledge
	Judging the Qualities of Things, Services, or People
	Guiding, Directing, and Motivating Subordinates
	Coordinating the Work and Activities of Others
	Resolving Conflicts and Negotiating with Others
	Coaching and Developing Others
	Provide Consultation and Advice to Others
	Developing and Building Teams
Low-skill activities	Controlling Machines and Processes
	Handling and Moving Objects
	Performing General Physical Activities
	Documenting/Recording Information
	Inspecting Equipment, Structures, or Material

Note. Work activities lacking a distinct skill polarity are categorized as neutral and are subsequently excluded from the analytical model because their relative importance varies substantially across occupations.

Table S7. Work activity classification divergences

Work Activity (O*NET)	Manual Categorization	ChatGPT-5 Classification	Gemini 2.5 Pro Classification
Documenting/Recording Information	Low-skill	Low-skill	Neutral
Inspecting Equipment, Structures, or Material	Neutral	Low-skill	Neutral
Estimating the Quantifiable Characteristics of Products, Events, or Information	Neutral	High-skill	Neutral
Monitoring Processes, Materials, or Surroundings	Neutral	Low-skill	Neutral
Monitoring and Controlling Resources	Neutral	High-skill	Neutral
Performing Administrative Activities	Neutral	Low-skill	Neutral
Staffing Organizational Units	Neutral	High-skill	High-skill
Communicating with People Outside the Organization	Neutral	High-skill	Neutral
Communicating with Supervisors, Peers, or Subordinates	Neutral	High-skill	Neutral
Establishing and Maintaining Interpersonal Relationships	Neutral	High-skill	Neutral
Interpreting the Meaning of Information for Others	Neutral	High-skill	High-skill
Performing for or Working Directly with the Public	Neutral	Low-skill	Neutral
Selling or Influencing Others	Neutral	High-skill	High-skill
Training and Teaching Others	Neutral	High-skill	High-skill
Evaluating Information to Determine Compliance with Standards	Neutral	High-skill	High-skill
Organizing, Planning, and Prioritizing Work	Neutral	High-skill	High-skill
Scheduling Work and Activities	Neutral	High-skill	Neutral
Drafting, Laying Out, and Specifying Technical Devices, Parts, and Equipment	Neutral	High-skill	High-skill
Repairing and Maintaining Electronic Equipment	Neutral	Low-skill	High-skill
Repairing and Maintaining Mechanical Equipment	Neutral	Low-skill	High-skill

Note. The O*NET database evolves to reflect labor market dynamics. To ensure longitudinal comparability with our historical baseline (O*NET 5.0, 2003), the item "Working with Computers" was excluded from our analysis due to differences in its data and emphasis over time. Accordingly, all agreement rate calculations and analyses are based on the remaining 40 core activities.

Table S8. Robustness of the economic effects of TSR across measurement paradigms

	AI Classifications		Conventional Indicator	Fixed O*NET base years		
	(1)	(2)	(3)	(4)	(5)	(6)
ASR	0.036***	0.031***	0.037***	0.034***	0.035***	0.035***
	(0.009)	(0.010)	(0.009)	(0.009)	(0.009)	(0.009)
TSR_gpt	0.071***					
	(0.021)					
TSR_gemini		0.126***				
		(0.033)				
TSR_traditional			0.022***			
			(0.008)			
TSR_2000				0.543***		
				(0.140)		
TSR_2010					0.417***	
					(0.124)	
TSR_2020						0.461***
						(0.135)
Constant	12.931***	12.948***	12.845***	12.566***	12.547***	12.514***
	(0.389)	(0.390)	(0.404)	(0.430)	(0.438)	(0.443)
Observations	1445	1445	1445	1445	1445	1445
Controls	Yes	Yes	Yes	Yes	Yes	Yes
Year fixed effects	Yes	Yes	Yes	Yes	Yes	Yes
City fixed effects	Yes	Yes	Yes	Yes	Yes	Yes
R^2	0.980	0.981	0.980	0.980	0.980	0.980

Note. TSR_gpt and TSR_gemini are derived from classifications of 40 O*NET work activities into high-skill, low-skill, and neutral categories by the ChatGPT-5 and Gemini 2.5 Pro models, respectively (*SI* Appendix Text 2). TSR_traditional is the ratio of high-skilled to low-skilled labor. Following official occupational classifications, high-skilled labor includes leaders of state organs, party and mass organizations, enterprises, public institutions, and professional/technical personnel. Low-skilled labor comprises the remainder of the workforce. To address concerns regarding temporal stability and database versioning, TSR_2000, TSR_2010, and TSR_2020 are constructed by fixing the occupational skill content to the O*NET baselines of the years 2000, 2010, and 2020, respectively.

Table S9. Parameters for standardization and compensation formula

Category	Parameter	Value	Description
Standardization parameters	μ_{ASR}	2.6274	Mean of raw ASR, sample 2000–2020
	σ_{ASR}	0.6249	Std. dev. of raw ASR, sample 2000–2020
	μ_{TSR}	0.7672	Mean of TSR, sample 2000–2020
	σ_{TSR}	0.1448	Std. dev. of raw TSR, sample 2000–2020
Regression coefficients	β_1	0.0385 (0.0092)	Effect of standardized ASR
	β_2	0.0903 (0.0196)	Effect of standardized TSR
	β_3	0.0173 (0.0061)	Interaction effect between ASR and TSR
Benchmark values (2020)	$ASR^*_{\mathrm{std},2020}$	-0.5895	Standardized ASR in 2020
	$TSR^*_{\mathrm{std},2020}$	0.9670	Standardized TSR in 2020

Note. All parameters are estimated based on the historical sample (2000–2020). Regression coefficients (β_1, β_2, β_3) are taken from Eq. 6. Robust standard errors are reported in parentheses. Benchmark values refer to observed standardized parameters in 2020, which serve as the reference point for the projection.

SI References

1. United Nations (2025) World Population Prospects 2024. Available at: https://population.un.org/wpp/ (accessed 23 January 2026).
2. Y. Kwak, Z. A. Pardos, Bridging large language model disparities: Skill tagging of multilingual educational content. *Brit. J. Educ. Technol.* **55**, 2039-2057 (2024). https://doi.org/10.1111/bjet.13465
3. Y. Wang *et al.*, Evaluating large language models as raters in large-scale writing assessments: A psychometric framework for reliability and validity. *Comput. Educ. Artif. Intell.* **9**, 100481 (2025). https://doi.org/10.1016/j.caeai.2025.100481
4. S. Bhandari, Y. Liu, Y. Kwak, Z. A. Pardos, Evaluating the psychometric properties of ChatGPT-generated questions. *Comput. Educ. Artif. Intell.* **7**, 100284 (2024). https://doi.org/10.1016/j.caeai.2024.100284
5. Y. Chang *et al.*, A survey on evaluation of large language models. *ACM Trans. Intell. Syst. Technol.* **15**, 1-45 (2024). https://doi.org/10.1145/3641289
6. National Bureau of Statistics of China (2026) National Economy Pushed Forward with Innovation-led and High-quality Development and Expected Targets Achieved Successfully in 2025. National Bureau of Statistics of China. Available at: https://www.stats.gov.cn/english/PressRelease/202601/t20260119_1962328.html (accessed 19 January 2026).
7. P. Rees, N. van der Gaag, J. de Beer, F. Heins, European regional populations: Current trends, future pathways, and policy options. *Eur. J. Popul.* **28**, 385-416 (2012). https://doi.org/10.1007/s10680-012-9268-z
8. M. T. Gastner, M. E. J. Newman, Diffusion-based method for producing density-equalizing maps. *Proc. Natl. Acad. Sci. U.S.A.* **101**, 7499-7504 (2004). https://doi.org/10.1073/pnas.0400280101
9. T. T.-L. Chong, L. Lu, S. Ongena, Does banking competition alleviate or worsen credit constraints faced by small- and medium-sized enterprises? Evidence from China. *J. Bank. Financ.* **37**, 3412-3424 (2013). https://doi.org/10.1016/j.jbankfin.2013.05.006
10. State Council of the People's Republic of China (2014) Notice on Adjusting the Classification Standards of Urban Scale. Available at: https://www.gov.cn/zhengce/content/2014-11/20/content_9225.htm (accessed 30 January 2026).
